# A detailed description of the development of the hemichordate *Saccoglossus kowalevskii* using SEM, TEM, Histology and 3D-reconstructions

**DOI:** 10.1186/1742-9994-10-53

**Published:** 2013-09-06

**Authors:** Sabrina Kaul-Strehlow, Thomas Stach

**Affiliations:** 1 Department für Integrative Zoologie, Universität Wien, Althanstr. 14, 1090, Wien, Austria; 2 Institut für Biologie, Humboldt-Universität zu Berlin, Philippstr. 13, 10115, Berlin, Germany

**Keywords:** Evolution of deuterostomia, Coelom formation, Left-right asymmetries, Hemichordates, 3D-reconstruction, Mesoderm development, Ultrastructure, Enterocoely, *Saccoglossus*

## Abstract

**Introduction:**

Traditionally, the origin of the third germ layer and its special formation of coelomic cavities by enterocoely is regarded to be an informative character in phylogenetic analyses. In early deuterostomes such as sea urchins, the mesoderm forms through a *single* evagination pinching off from the apical end of the archenteron which then gives off mesocoela and metacoela on each side. This echinoid-type coelom formation has conventionally been assumed to be ancestral for Deuterostomia. However, recent phylogenetic analyses show that Echinodermata hold a more derived position within Deuterostomia. In this regard a subgroup of Hemichordata, namely enteropneusts, seem to host promising candidates, because they are supposed to have retained many ancestral deuterostome features on the one hand, and furthermore share some characteristics with chordates on the other hand. In enteropneusts a wide range of different modes of coelom formation has been reported and in many cases authors of the original observations carefully detailed the limitations of their descriptions, while these doubts disappeared in subsequent reviews. In the present study, we investigated the development of all tissues in an enteropneust, *Saccoglossus kowalevskii* by using modern morphological techniques such as complete serial sectioning for LM and TEM, and 3D-reconstructions, in order to contribute new data to the elucidation of deuterostome evolution.

**Results:**

Our data show that in the enteropneust *S. kowalevskii* all main coelomic cavities (single protocoel, paired mesocoela and metacoela) derive from the endoderm via enterocoely as *separate* evaginations, in contrast to the aforementioned echinoid-type. The anlagen of the first pair of gill slits emerge at the late kink stage (~96 h pf). From that time onwards, we documented a temporal left-first development of the gill slits and skeletal gill rods in *S. kowalevskii* until the 2 gill slit juvenile stage.

**Conclusions:**

The condition of coelom formation from *separate* evaginations is recapitulated in the larva of amphioxus and can be observed in crinoid echinoderms in a similar way. Therefore, coelom formation from *separated* pouches, rather than from a *single* apical pouch with eventual subdivision is suggested as the ancestral type of coelom formation for Deuterostomia. Left-right asymmetries are also present in echinoderms (rudiment formation), cephalochordates (larval development), tunicates (gut coiling) and vertebrates (visceral organs), and it is known from other studies applying molecular genetic analyses that genes such as *nodal*, *lefty* and *pitx* are involved during development. We discuss our findings in *S. kowalevskii* in the light of morphological as well as molecular genetic data.

## Introduction

Deuterostomia comprise one of the major branches of Bilateria, including the higher taxa Echinodermata (sea urchins, sea stars, sea lilies, sea cucumbers, brittle stars), Hemichordata (pterobranchs and enteropneusts) and Chordata (amphioxus, tunicates and vertebrates) [1-3]. There are two apomorphies by which Deuterostomia is supported. Eponymous for this group is deuterostomy, meaning the blastoporus forms the definitive anus, while the mouth opening develops secondarily [3,4]. Also, the origin of the third germ layer, the mesoderm, is remarkable as the prospective coelomic cavities derive from endodermal evaginations pinching off of the archenteron. This process is called enterocoely. In principle, two modes of coelom formation can be distinguished in triploblastic animals and have been associated with one of the main animal groups: schizocoely in protostomes and enterocoely in deuterostomes [5]. The suggestion of homology of the third germ layer across the animal kingdom can be traced back to the early studies of Hertwig and Hertwig [6] and Mastermann [7] and has found support in some recent studies [5,8-10]. Therefore, the origin of the mesoderm and its special formation including a coelomic cavity has traditionally been given high importance in phylogenetic analyses [11-13]. Although the above mentioned classification is applicable in many cases, exceptions have been reported as well (e.g. see [14-16]). Some of the oldest and most elaborate studies on the development of the mesoderm in deuterostomes were conducted on members of Echinodermata [17-22]. In echinoids it has been reported that a single, voluminous sac pinches off from the anterior end of the endoderm or archenteron, which eventually subdivides to give off paired axo-, hydro-, and somatocoel (corresponding to pro-, meso-, and metacoel in enteropneusts) on each side (for reviews see [3,23]). Because Echinodermata has classically been placed as the earliest branching deuterostome group [4,24,25], it is concluded that this echinoid-type mode of coelom formation represents the ancestral condition for Deuterostomia, although studies show differences even between echinoderm classes [17,26]. Most of the recent molecular phylogenetic and combined analyses support a sister group relationship between Echinodermata and Hemichordata, together comprising Ambulacraria [27-32]. Ambulacraria in turn is supposed to be the sister group to Chordata. Some authors argue that Xenacoelomorpha (Xenoturbellida + Acoelomorpha) may represent another deuterostome group [33], but see for example Hejnol et al. [34] or Edgecombe et al. [2] who support a classical view of placing them more basal within Bilateria. The Ambulacraria hypothesis has furthermore been supported by genetic, morphological, and paleontological evidence e.g. [35], and accordingly the view of deuterostome evolution has changed. Given that Echinodermata are no longer considered as the earliest branching deuterostome taxa, conclusions on deuterostome evolution and character polarity have to be carefully re-evaluated. Currently, the reconstruction of the last common ancestor of Ambulacraria and even Deuterostomia varies greatly depending on the chosen phylogenetic hypothesis. On the one hand, phylogenetic analyses show Pterobranchia and Hemichordata as sister groups [3,30,36] and therefore postulate a pterobranch-like ancestor of Ambulacraria. On the other hand, other authors place pterobranchs within the Enteropneusta [37-39]. Following the latter ones, enteropneust hemichordates seems to be promising in elucidating ambulacrarian and deuterostome evolution, because these animals have supposedly retained many ancestral deuterostome characters [35,38,40]. For instance, they are solitary, marine, free-living, and worm-like animals with a comparably simple bilaterally symmetric body plan and a biphasic life-cycle featuring a planktonic tornaria larva [30,41]. Furthermore, enteropneusts exhibit features with some similarities to chordates (gill slits, internalized neural tube, stomochord, collagenous gill bars), probably due to common heritage [35,42,43].

The formation of coelomic cavities (pro-, meso-, and metacoel) in enteropneusts has been studied several times [41,44-48]. Despite the fact that enteropneusts compose a group of quite phenotypically homogenous animals, early researchers reported at least five different modes of coelom formation, which have since been depicted throughout textbooks or treatises [3,11,49]. However, occasionally the interpretations drawn from the original light-microscopical data were based on a very limited set of stages (see also comments in [30]). Moreover, in determining germ layer affiliation of early embryonic tissues, it is of crucial importance to identify and trace the extracellular matrix (*ecm*) separating tissues accurately, as it forms a basal lamina on which epithelial cells rest on [14,15,50-52]. The thickness of such *ecm* in marine invertebrate embryos is often below the resolution distance of classical light microscopy (< 0.2 μm), a potential explanation for the different results of the classical comparative studies. A re-examination of coelom formation in hemichordates with modern techniques using electron microscopy is lacking until today, as the latest detailed morphological studies on the ontogeny of enteropneusts were conducted in the 1950s [53].

The aim of the present study is to fill this gap. We investigated densely sampled successive ontogenetic stages of the direct-developing enteropneust *Saccoglossus kowalevskii* using SEM, TEM, and histology. The accurate analysis of complete serial sections in combination with computer-aided 3D-reconstructions of all major organ systems exemplifies the detailed illustration of a profound description of the development of an enteropneust.

Our data show, that all of the five main coelomic cavities arise from the endoderm by way of enterocoely. Moreover, the mesocoela and metacoela are given off as separated coelomic pouches from the middle and posterior regions of the endoderm, respectively. By re-evaluating the quality of previous reports on coelom formation in other enteropneusts, we found some discrepancies and therefore argue that a re-investigation using modern methods is warranted in other cases. By comparing our results with those reported from other deuterostomes such as echinoderms, pterobranchs, and cephalochordates, we argue that instead of an echinoid-type coelom formation, it is more likely that coelom formation in the last common ancestor of Deuterostomia was by a single anterior protocoel and paired meso- and metacoela evaginating from the middle and posterior regions of the endoderm, respectively. Consequently, the aforementioned echinoid-type of coelom formation has to be regarded as a derived condition that evolved within echinoderms.

## Results

### Early blastula stage

**Figure 1:**
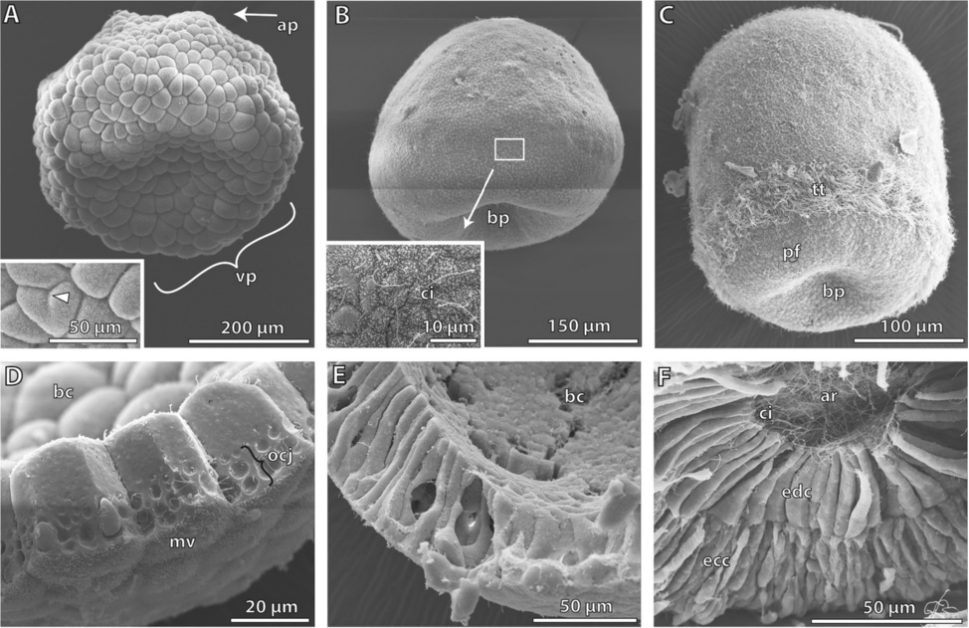
**Scanning electron micrographs showing different early embryological stages of *Saccoglossus kowalevskii *. (A)** The 8 hour post fertilization (h pf) blastula shows a prominent depression at the vegetal pole (*vp*). Inset shows blastomeres at higher magnification (high mag). Partial constrictions demonstrate ongoing cleavage (*arrowhead*). **(B)** The flattened gatrula (24 h pf) shows the invagination of the archenteron at the blastoporus region (*bp*). Inset: a single cilium (*ci*) is developed on each cell‘s surface. **(C)** Around 30 h pf gastrulation is completed. Embryos display an elongated shape. The multiciliated telotroch (*tt*) is developed about 50 μm from the blastopore. **(D)** The cells of the blastula stage are of cuboid shape, and covered apically with microvilli (*mv*). The cells are interconnected by occluding cell junctions (*ocj*). **(E)** The cells of the gastrula are highly prismatic enclosing the blastocoel (*bc*). **(F)** The blastocoel is reduced by the endodermal germ layer almost completely. Endodermal cells (*edc*) are columnar, bordering the lumen of the archenteron (*ar*) with their apical cell surface. Long cilia protrude into the lumen. Ectodermal cells (*ecc*) exhibit a columnar shape, with sometimes very narrow elongated parts, conveying a pseudostratified appearance. *ap* animal pole, *pf* perianal field.

The fertilized eggs of *Saccoglossus kowalevskii* develop into an early blastula stage measuring about 400 μm in height and 430 μm in breadth at the age of 8 h post fertilization (pf) (Figure 1A,D). The early blastula exhibits a somewhat irregular spherical shape, slightly punched in at the vegetal pole. A shallow notch at one side characterizes the vegetal pole of the embryo, indicating the position of the prospective blastopore (Figure 1A). Under the scanning electron microscope the individual cells comprising the early blastula can clearly be distinguished from each other. On some cell surfaces partial constrictions indicate ongoing cleavage processes (Figure 1A inset). The cells at this developmental stage are more or less of elongated cuboid shape, measuring about 20 μm in diameter and 30 μm in height ranging from the internal surface facing the blastocoel inside the embryo to the outer surface (Figure 1D). The outer cell surface is covered with evenly distributed short microvilli. Furthermore, about 6 μm of the space underneath the apical cell surface is occupied by an area comprising occluding cell junctions (Figure 1D). In particular, these cell junctions are supposed to interlink the neighbouring cells.

### Early gastrula stage

During the next few hours the so-called early or flattened gastrula is formed by repetitive cleavages of the cells (Figure 1B). The beginning of gastrulation is indicated by the invagination of the blastomere cells in the centre of the flattened vegetative pole, forming the opening of the blastopore. After 24 h pf, the early gastrula is cup-shaped and measures 300 μm in height and 340 μm in width (Figure 1B). Moreover, the cells now have a different shape they are highly columnar measuring 60 μm in length and barely 8 μm in width (Figure 1E). They are tall and form a smooth outer as well as inner blastocoel-facing surface. A close-up of the outer cell surface shows that the majority of the cells have developed a single cilium between the dense microvilli. In the region of the prospective telotroch notably longer cilia are present (Figure 1B inset). At this stage of development the embryos are constituted of one single layer of cells enclosing an inner lumen namely, the blastocoel.

### Gastrula stage

At an age between 30 and 36 h pf most embryos develop an elongated, ovoid shape and measure about 300 μm in length (Figure 1C). One of the most important developmental characters is the conspicuous telotroch. The telotroch is constituted of multiciliated cells encircling the animals approximately 50 μm anterior to the blastopore. The remaining cells of the ectoderm are monociliated (Figure 1C). A SEM micrograph of a dissected animal reveals the diploblastic organization of the embryos at this stage of development (Figure 1F). The invaginated endoderm tightly adjoins the ectoderm, no space remains between the two germ layers, thus obliterating the blastocoel almost completely. The ectoderm consists of a highly columnar epithelium. The shape of the cells is somewhat irregular. Sometimes, cells exhibit slender elongations giving the whole epithelium a pseudostratified appearance (Figure 1F). The ectodermal cells measure about 30 μm in height. Their bulged surfaces indicate a cytoplasm rich in vesicles. The majority of these vesicles at this stage of development contain yolk. The endodermal cell layer likewise contains a monolayered epithelium measuring about 40 μm in height. The cells have a columnar shape, bordering a central lumen along their apical cell surface, the lumen of the archenteron. The apical cell surfaces of the endodermal cells are unevenly covered with short microvilli. Moreover, each endodermal cell is equipped with a single cilium which protrudes into the lumen of the archenteron (Figure 1F).

### 1st groove stage

#### Gross morphology

**Figure 2:**
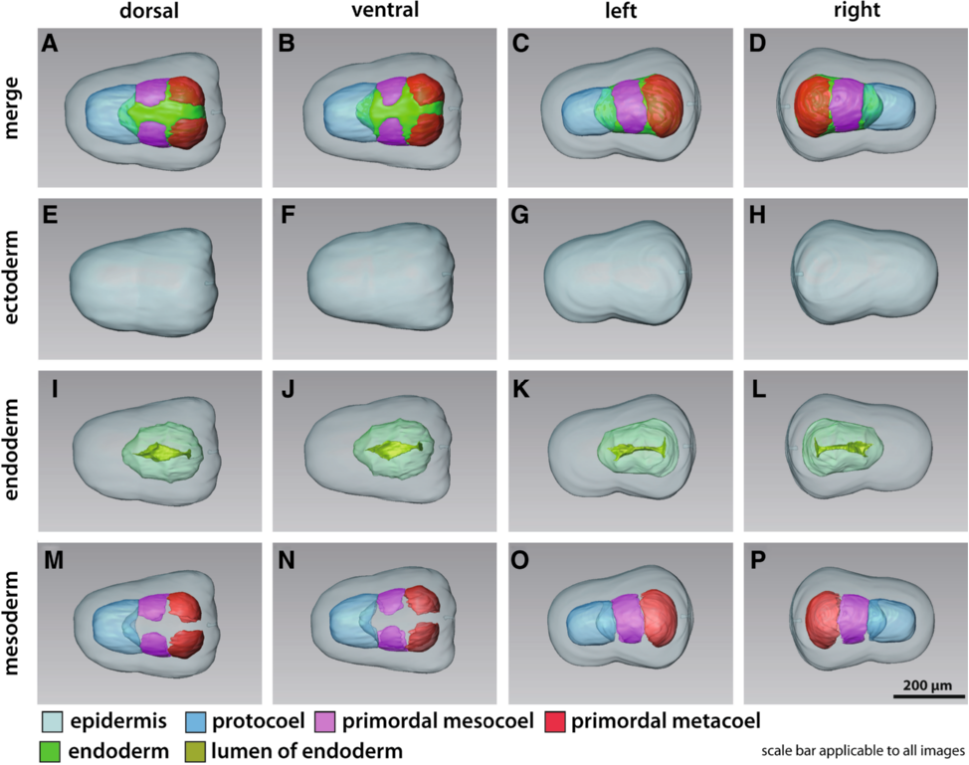
**3D reconstruction of the embryo of *Saccoglossus kowalevskii *at 36 h pf.** Rows from left to right: dorsal, ventral, left, and right view. Columns from top to bottom: The merge row **(A-D)** shows the embryo with all reconstructed structures. Ectoderm row **(E-H)** shows external shape of embryo, telotroch not shown. Endoderm row **(I-L)** reveals the transparent endodermal tissue (*light green*) and the position of the endodermal lumen (*yellowish green*). Mesoderm row **(M-P)** shows the position of the anterior protocoel (*blue*) and the primordal meso- (*pink*) and metacoelic (*red*) tissue. Download interactive 3D-PDF as Additional file 1.

**Figure 3:**
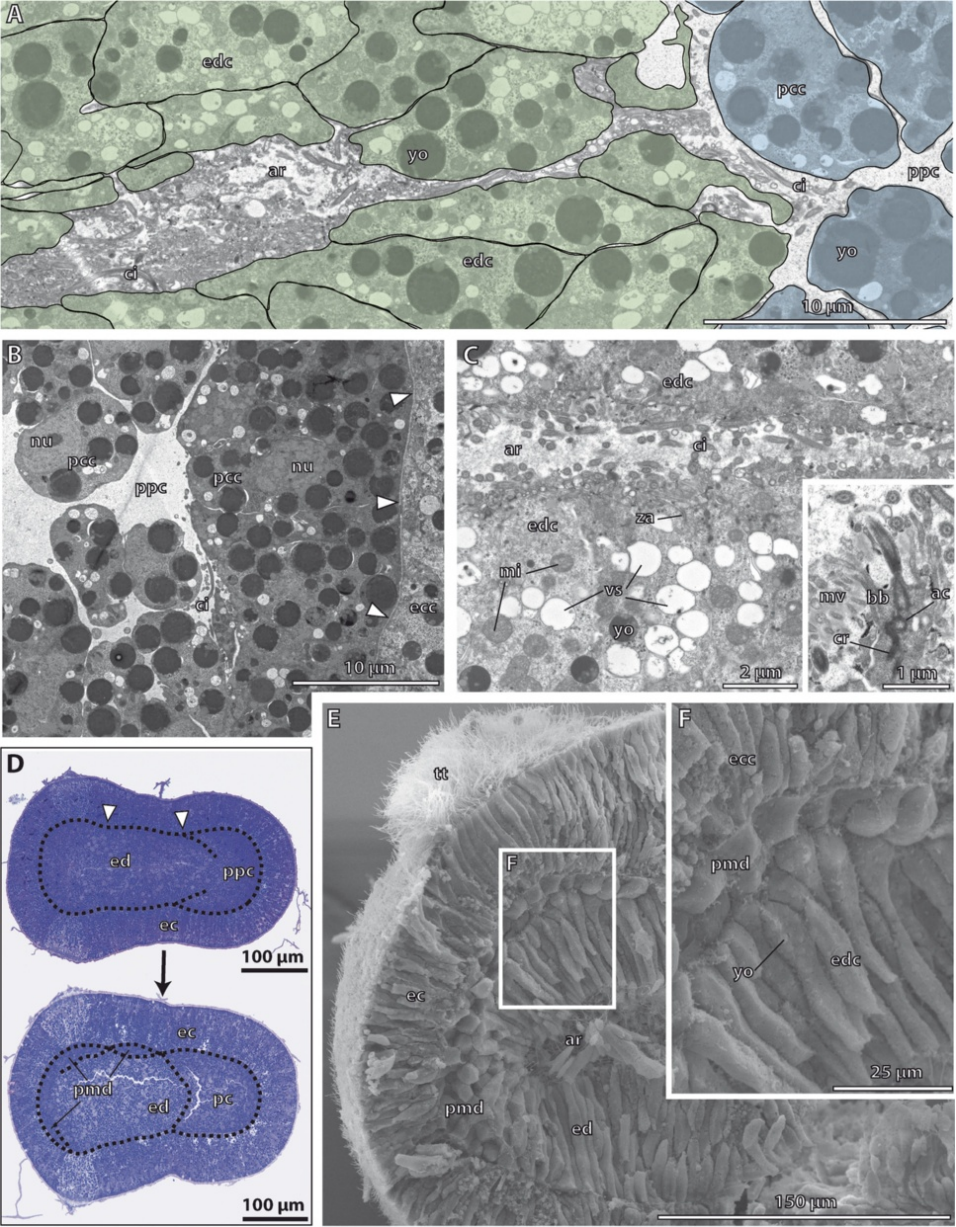
**Histology and fine structure of the embryo of*Saccoglossus kowalevskii*at 36 h pf.A - C**, Electron micrographs. **D** Semithin sagittal sections. **E-F** Scanning electron micrographs. **(A)** The lumen of the archenteron (*ar*) is still continuous with the anterior primordal protocoel (*ppc*). **(B)** The apical cell processes of the primordal protocoelic cells (*pcc*) are goblet-shaped and their basal portion is resting on ecm (*arrowheads*) separating the protocoel from the ectoderm (*ec*). **(C)** The epithelial endodermal cells (*edc*) are filled with several vesicles (*vs*) apically. Inset: Each cilium is connected to the cytoplasm by an anchoring complex. **(D)** Sagittal sections of two specimens. At the onset of mesoderm formation, the endoderm (*ed*) shows two shallow constrictions (*arrowheads*) mirroring the embryo’s future tripartite body organization. The lower embryo is slightly older indicated by the completely separated protocoel (*pc*). The primordal mesoderm (*pmd*) starts to establish laterally, at the middle and posterior regions of the endoderm. The extracellular matrix (*ecm*) is indicated by the dotted line. **(E**-**F)** Sagittally dissected embryo showing the internal organization of the posterior end (note the position of the telotroch (*tt*)). Ectoderm and endoderm have close contact to each other. A third layer of cells, the primordal mesoderm, reaches between ecto- and endoderm. *ac* accessory centriole, *bb* basal body, *ecc* ectodermal cell, *ci* cilium, *cr* ciliary rootlet, *mi* mitochondrion, *mv* microvilli, *nn* nerve net, *yo* yolk, *za* zonula adherens.

The embryos at the 1st groove stage are of elongated shape measuring 450 μm in length, with a more rounded anterior end and a more flattened posterior end (Figure 2A-P, and Figure 3D,E Additional file 1). The conspicuous telotroch surrounding the embryo separates the posterior perianal field from the larger anterior part of the animal. The telotroch is constituted of multiciliated cells (Figure 3E). This developing stage is named after a shallow circular groove that is visible from the exterior ca. 200 μm posterior to the anterior pole (Figures 2, and 3D). This groove characterizes the boundary between the prospective proboscis and collar region. Later on, it constricts deeply to separate the proboscis from the remaining body. SEM of dissected embryos and serial sections reveal the internal anatomy.

The endoderm extends to the posterior end of each embryo and is separated from the ectoderm by means of a basement membrane, except in the blastoporus region (supplementary (S) Additional file 2: Figure S2D). Here, ectodermal and endodermal tissues come in direct contact to each other. The endodermal lumen is a flattened tube, measuring hardly more than 2 μm in height, but up to 50 μm in width in the prospective collar region (Figure 2I,J). These lateral protrusions of the endodermal wall are the anlagen of the pouches of the prospective mesocoela.

The anterior portion of the endodermal tissue will give rise to the anteriormost mesodermal cavity, i.e. the protocoel (Figure 2A-D, M-P, and Figure 3D). In most of the examined embryos at this stage, the protocoelic cavity is still continuous with the lumen of the endoderm (Figure 3A), although laterally the basement membrane begins to constrict the protocoel from the remaining endoderm (Additional file 2: Figure S2F,G). In the 3D reconstruction of a slightly further developed embryo the protocoel is completely separated from the remaining endoderm, as it forms the ontogenetically first completely separated coelomic cavity.

In contrast, the prospective meso- and metacoela form lateral pouches in the middle and posterior regions of the endoderm, respectively (see Figure 2A-D and M-P for coloured primordal mesodermal cells). Both mesodermal anlagen constitute left and right parts which later on surround the endoderm.

#### Fine structure of the endoderm

The endoderm consists of a highly columnar monociliated epithelium, which encloses a slit-like central lumen, the archenteron (Figure 3A,C,E,F). The central lumen extends from the anterior region, where it connects to the protocoelic cavity, to the posterior region, where it bifurcates dorsally and ventrally (Figure 2K,L). At the anterior margin of the endoderm, the protruding axonemata of the cilia of the endodermal cells project into the prospective protocoelic cavity that is still continuous with the endodermal lumen at this 1st groove stage (Figure 3A). The endodermal cells measure about 20 μm in height and border the luminal side with narrow elongations about 3 μm in breadth (Figure 3E,F, Additional file 2: Figure S2D,E). The apical surface of each endodermal cell is equipped with numerous slender microvilli measuring 1 μm in height and 100 nm in width. The microvilli are evenly distributed and encircle a single cilium to form a collar-like arrangement (Figure 3C inset). The axoneme of each cilium shows a regular 9 × 2 + 2 microtubular pattern in cross sections, which is common for motile cilia. The cilia are anchored into the apical cytoplasm by one striated rootlet running basally into the cell body (Figure 3C inset). Neighbouring cells are interconnected apically by adherens junctions (Figure 3C). The apical cytoplasm is often filled with many electron-opaque vesicles of various diameters measuring between 400 nm and 950 nm in diameter (Figure 3C). The high number of vesicles indicates a secretory function as expected for endodermal tissue. Moreover, different granules are present within the cytoplasm, giving it a moderately electron-dense appearance. The nucleus has a central position and contains a spherical and electron-dense nucleolus (Additional file 2: Figure S2D). Mitochondria are numerous and located in the distal half of the cell between the cell surface and the nucleus. Few yolk granules are found in the apical cell portion. In contrast, yolk granules of various diameters represent the predominating type of organelles in the central and basal compartments of the cytoplasm (Figure 3D,F, and Additional file 2: Figure S2D,F). The high amount of yolk within all endodermal cells reflects the developmental stage of these embryos. At their bases, the endodermal cells attach to a basement membrane, which separates endoderm from ectoderm.

#### Fine structure of the protocoel

Almost the complete protocoel is packed with rather undifferentiated protocoelic cells, except for a limited central fluid-filled space, the prospective protocoelic cavity (Figure 3A, B). The shape of these cells is less columnar, compared to those of the endodermal cells. The protocoelic epithelium consists of stalked goblet-shaped cells up to 80 μm long, with a spherical somatic part projecting into the lumen of the protocoel (Figure 3A, B, and Additional file 2: Figure S2A, C). A narrow and elongated neck region connects the somatic part to the more voluminous basal portion that is attached to the basement membrane by hemidesmosomes. The nucleus is generally located within the goblet-shaped region of the cell. The cytoplasm contains numerous spherical and electron-dense yolk granules, as well as other organelles. The protocoelic cells posses a single cilium at their apical cell surface. The remaining surface is smooth, no microvilli are present.

#### Fine structure of the meso- and metacoel

Light microscopic sections show a slightly darker staining in those areas, where the collar and trunk mesoderm will differentiate (Figure 3D). Scanning electron micrographs show the prospective trunk mesoderm, i.e. the metacoel developing as a third germ layer between the ectoderm and endoderm (Figure 3E,F inset). The trunk mesoderm consists of two layers of more or less cuboid cells measuring about 12 μm in length and width. The surface of the mesodermal cells is smooth and comparable to those of the endodermal cells, but it remains unclear with SEM if cilia are developed at this stage.

### Early kink stage

#### Gross morphology

**Figure 4:**
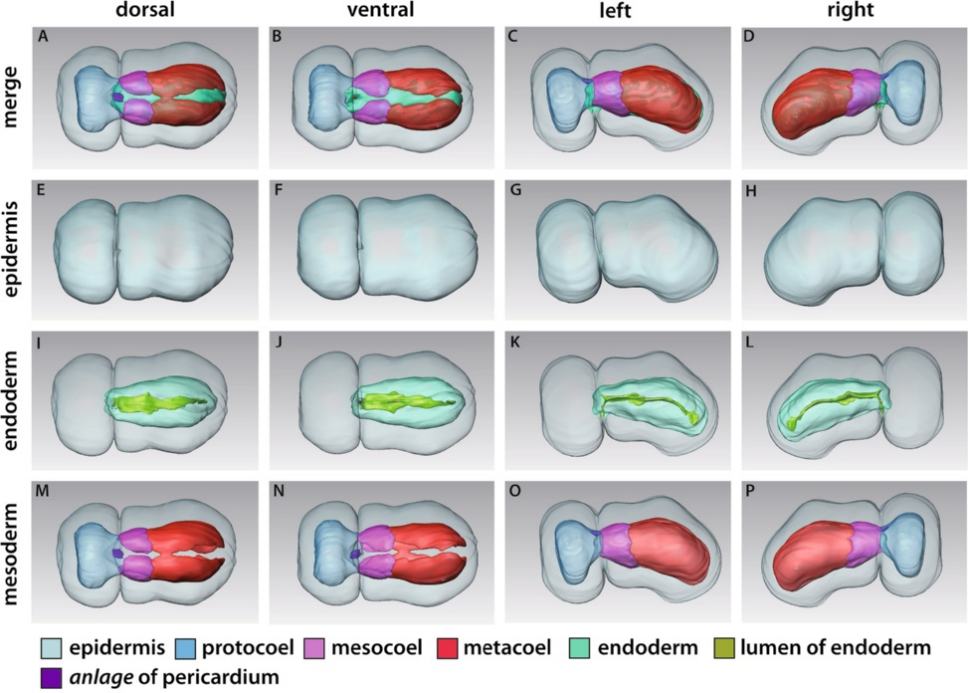
**3D reconstruction of the embryo of *Saccoglossus kowalevskii *at early kink stage (~ 56 h pf).** Rows from left to right: dorsal, ventral, left and right view. Columns from up to down: The merge row **(A-D)** shows the embryo with all reconstructed structures. Epidermis row **(E-H)** shows the external shape of embryo. The telotroch is not shown. Endoderm row **(I-L)** reveals the transparent endodermal tissue (*light green*) and the straight course of the endodermal lumen (*yellowish green*). Lateral evaginations (*double arrowheads*) in the collar region illustrate the connections to the pockets that constitute the mesocoela. Mesoderm row **(M-P)** shows the position of the anterior protocoel (*blue*) and the paired meso- (*pink*) and metacoelic (*red*) compartments. The anlage of the pericardium (*purple*) emerges in the posteriormost region of the proboscis (*pr*) within the ectoderm. Download interactive 3D-PDF as Additional file 3. *co* collar, *mo* mouth opening, *stA* anlage of the stomochord, *tr* trunk.

**Figure 5:**
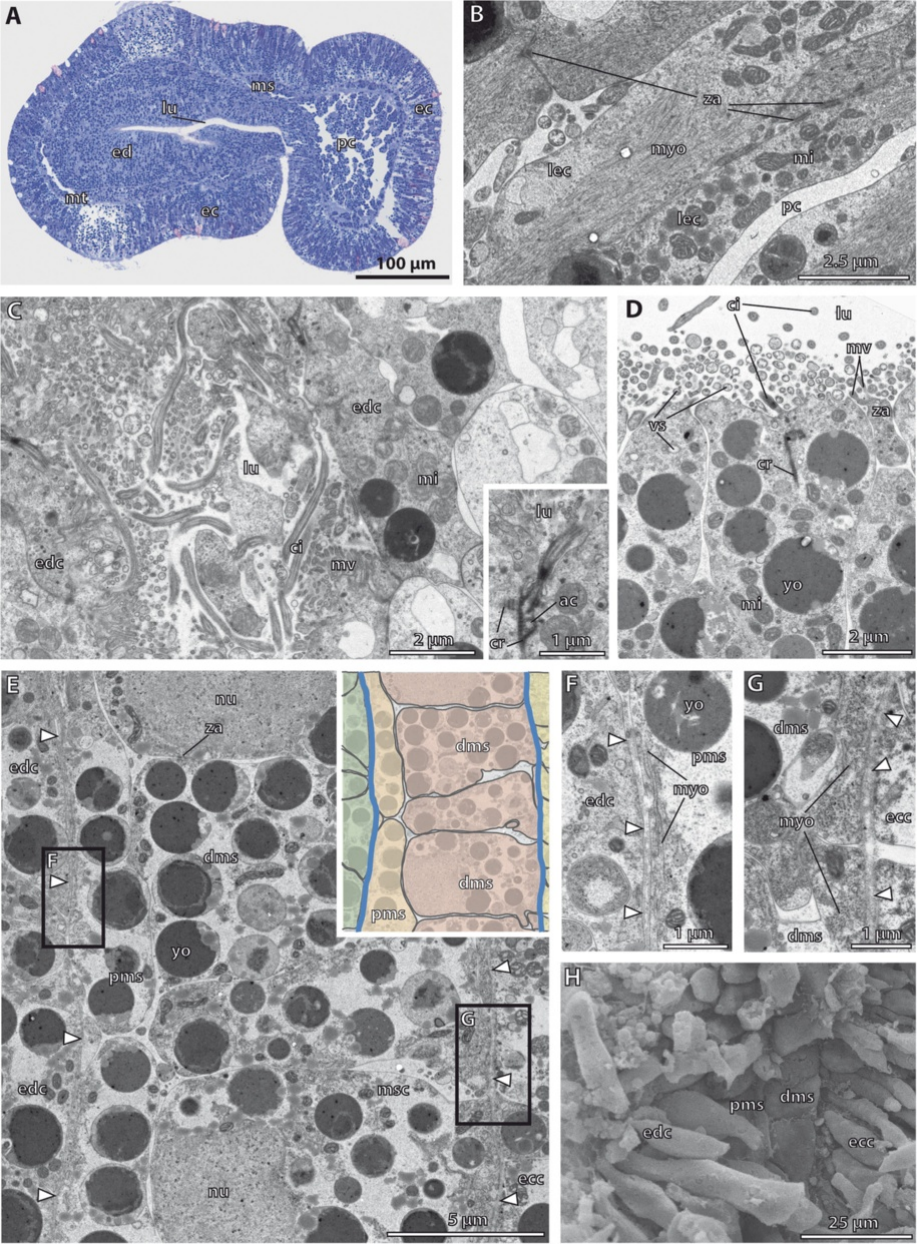
**Histology and fine structure of the early kink stage of *Saccoglossus kowalevskii*. (A)** Semithin sagittal section showing the inner organization of the embryo. The lateral evagination of the endodermal wall constitutes the rudiment of the earlier connection to the mesocoela. Fine structure of the tubular connection is shown in C. **(B)** The longitudinal epitheliomuscle cells (*lec*) of the protocoel (*pc*) are rich in myofilaments (*myo*) and mitochondria (*mi*). Neighbouring cells are interconnected by zonulae adherentes (*za*). **(C)** The cells comprising the former connection to the mesocoela are monociliated and resemble those of the endodermal lining. Inset: Each cilium (*ci*) is anchored to the cytoplasm by a short horizontal rootlet and a second vertical rootlet. **(D)** The endodermal cells (*edc*) are columnar and monociliated. They are covered by numerous bulbus microvilli (*mv*) and several yolk granules (*yo*) are present within the cytoplasm. **(E)** Cross section of the mesocoelic mesoderm composed of two layers. The proximal mesothelium (*pms*) is made up of flattened cells, while the distal mesothelium (*dms*) contains more cuboid and voluminous cells. Note the course of the ecm (*arrowheads*). **(F)** The proximal flattened cells contain basal myofilaments in a circular arrangement. There is a virtual lumen present between both cell layers as indicated by interspersed cilia (see S2D for high mag). **(G)** The distal cells contain basal myofilaments in a longitudinal arrangement. **(H)** SEM of a dissected embryo showing the same area as seen in E. The mesodermal is composed of a distal and proximal layer of cells. *ac* accessory centriole, *cr* ciliary rootlet, *ec* ecdoderm, *ecc* ectodermal cell, *ed* endoderm, *lu* lumen, *ms* mesocoel, *msc* mesodermal cell, *mt* metacoel, *nu* nucleus, *vs* vesicles.

Embryos at this developmental stage show a proboscis that is more flattened at the anterior end and is separated by a deep constriction from the rest of the body, the collar and trunk region (Figures 4A-H and 5A, Additional file 3). The animals measure about 450 μm in total length. The trunk region is elongated measuring up to 300 μm in length, and shows a slightly ventral kink.

The endoderm is present as a simple, blind-ending structure extending from the anterior collar region to the posterior end of the trunk. No anus is opened yet (Figures 4I-L, and 5A). However, the ventromedian mouth opening has developed to connect the anterior endoderm with the outer environment. The mouth opening is located exactly within the circular groove that separates the proboscis from the collar region (Figure 4K,L). The epithelium of the endoderm is about 45 μm high and surrounds the narrow slit-like lumen (Figures 4I-L and 5A and Additional file 4: Figure S4A). This lumen is flattened dorso-ventrally and follows a straight course through the embryo. Following the kink of the animal, it is slightly bent within the posterior trunk region. The endodermal wall shows a pair of lateral evaginations visible just anterior to the posterior margin of the collar region (Figures 4I,J and 5A). These are the former connections to the mesocoelic pouches, which derive from the archenteron via enterocoely. Beside the lateral evaginations, the endodermal wall shows a shallow longitudinal depression at the ventral midline within the collar region (Figure 4J-L). Moreover, the lumen of the gut sends a 20 μm short projection towards the anterodorsal region, representing the anlage of the stomochord (Figure, 4K and L).

The proboscis region is completely dominated by the anterior body cavity, the protocoel (Figures 4A-D, M-P, and 5A). At the lateral and dorsal sides the protocoel extends slightly posteriorly contacting the mesocoela at the margin of the collar region (Figure 4A,B,M,N). The 3D reconstruction shows a small, more or less triangulate mass of ectodermal cells within the ectoderm, dorsally above the posterior end of the protocoel. This is the anlage of the pericardium, that later forms a contractile fluid-filled coelomic cavity (see also [15] Figures 2A and 3A).

The paired mesocoelic and metacoelic pouches have expanded and extended in all directions to adjoin each other in antero-posterior direction and to encircle the median endoderm (Figure 4A-D, M-P). All coelomic sacs are separated from the endoderm by means of a thin sheath of *ecm* (Figure 5A,E and Additional file 4: Figure S4E). The middle pair of mesodermal pouches, the prospective mesocoela extend about 100 μm in length and demarcate the position of the collar region. The paired metacoel is restricted to the trunk region and encircles the posterior endoderm. The left and right cavities extend over about 280 μm in total length and are separated dorsally as well as ventrally by mesenteries.

#### Fine structure of the endoderm

The anterior end of the endoderm is flattened and protrudes shortly into the protocoel (Figures 4I-L and 5A). The endoderm is a highly columnar epithelium that appears to be pseudostratified in ultrathin cross sections. The endodermal cells measure about 65 μm in height but only 3.5 μm in width (Figure 5D and Additional file 4: Figure S4A) and border a central ellipsoid-shaped lumen located in the centre. Each cell is equipped with a single cilium and moreover numerous short microvilli measuring about 1 μm in height. Between microvilli numerous vesicles are seen apically in longitudinal sections. These vesicles are filled with different content (Figure 5D). The cilium shows a typical 9×2 + 2 microtubular arrangement and is anchored in the apical cytoplasm via a vertical striated rootlet. Neighbouring cells are interconnected by adherens junctions. The slightly elongated nucleus is central in position (Additional file 4: Figure S4) and contains an electron-dense, spherical nucleolus. Numerous yolk granules varying between 0.5 to 3 μm in diameter dominate the cytoplasm of endodermal cells. In general, the endodermal cells appear quite undifferentiated at the early kink stage. The conspicuous and abundant clear vesicles observed in the 1st groove stage (see Figure 3C) are no longer present (Figure 5D, Additional file 4: Figure S4). While endodermal cells are exclusively monociliated, the ectodermal epithelium consists of multiciliated supporting cells and different monociliated glandular cells. Those endodermal cells lining the former connection to the mesocoelic pouches are likewise monociliated cells equipped with numerous microvilli across the cell surface (Figure 5C, inset). The cilia are at least 5 μm long and anchored into the cytoplasm by two striated rootlets. The first rootlet remains short and runs horizontally, while the second one projects, slightly obliquely in vertical direction. The accessory centriole is attached to the vertical rootlet by means of a bundle of filaments (Figure 5C inset).

#### Fine structure of the protocoel

The protocoelic lining is composed of monociliated myoepithelial cells, which can be classified into two types, according to the different amount and the arrangement of myofilaments. A sagittal section of the proboscis shows that the cells positioned laterally in the protocoel have developed into long, slender cells, which span the protocoelic cavity completely in anterior-posterior direction (Figure 5B and Additional file 4: Figure S4B). These cells have a minimum length of 50 μm by measure barely 2 μm in width. They are connected to the basement membrane anteriorly as well as posteriorly by hemidesmosomes. The protocoelic lining cells are interconnected among each other by adherens junctions that are commonly found close to the more voluminous basal part of the cells (Figure 5B and Additional file 4: Figure S4B). Most of the cytoplasm of these cells is filled with myofilaments accompanied by numerous mitochondria. Myofilaments are orientated in longitudinal direction. The nucleus is of elongated shape and is located aside the myofilaments within the narrow elongated part of the cells. These slender longitudinal epitheliomuscle cells alternate with a second type of cells which contains basal myofilaments that are circularly arranged (Additional file 4: Figure S4B). The shape of these cells is less elongated. Instead, these cells appear more compact and ellipstic when examined in sagittal sections. These cells have a more electron-dense cytoplasm. The nucleus is located almost basally or is found at least in the basal portion of the cells. Besides the obligate presence of yolk granules, the cytoplasm contains several weakly electron-dense vesicles of various diameters. A sagittal section reveals that here the cells are less differentiated (Figure 5A). These cells retain the club- or goblet-shaped profile already described for the 1st groove stage. There may be individual bundles of circularly arranged myofilaments present in the very basal part of the cytoplasm.

#### Fine structure of the meso- and metacoel

Ultrastructural investigations show that the mesodermal tissue is composed of two layers (Figure 5E-H). This applies to the mesocoelic mesoderm as well as for the metacoelic mesoderm. There is no difference in ultrastructure between the coelothelia bordering both coelomic compartments. However, cross sections demonstrate that both cell layers rest on different basement membranes (Figure 5G, inset, F-G and Additional file 4: Figure S4E). The distal cells beneath the ectoderm display a cubical to columnar shape, measuring 11 μm in height and between 3 to 7 μm in width (Figure 5E, inset). The cytoplasm is electron light and contains several yolk granules the larger ones of which are more osmiophilic. Some mitochondria are observed throughout the cytoplasm. The nucleus is more or less spherical measuring 5 μm in diameter and is restricted to the apical part of the cells (Figure 5G). Myofilaments are present in the very basal portion of the cells. These filaments are about 15 nm in diameter and longitudinally arranged in all cells in cross sections (Figure 5G). The proximal layer of cells is resting on a very thin sheath of *extracellular matrix* (*ecm*) that separates the mesoderm from the endoderm (Figure 5E, inset). At some points this sheath becomes so narrow that it is discontinuous in the electron micrographs (Figure 5F). The cells of this layer consist of much more flattened cells, measuring only 2.5 μm in height but at least 13 μm in breadth. The cytoplasm is similar to the cytoplasm of the distal cells in most aspects. Distinctive characters are more flattened nuclei and the myofilaments, which are circularly arranged (Figure 5F).

Both cell layers show signs of epithelialisation and surround a virtual central lumen between them (Additional file 4: Figure S4D). Following serial sections, single cilia can be found at the apical cell surfaces, whose axonemata protrude into the virtual lumen (Figure 5E, Additional file 4: Figure S4D). Apical cell junctions and spot desmosomes are scarcely found and poorly developed. However, apical cell junctions are restricted to connect neighbouring cells of the same cell layer, but are never present between proximal and distal cells.

### Late kink stage

#### Gross morphology

**Figure 6:**
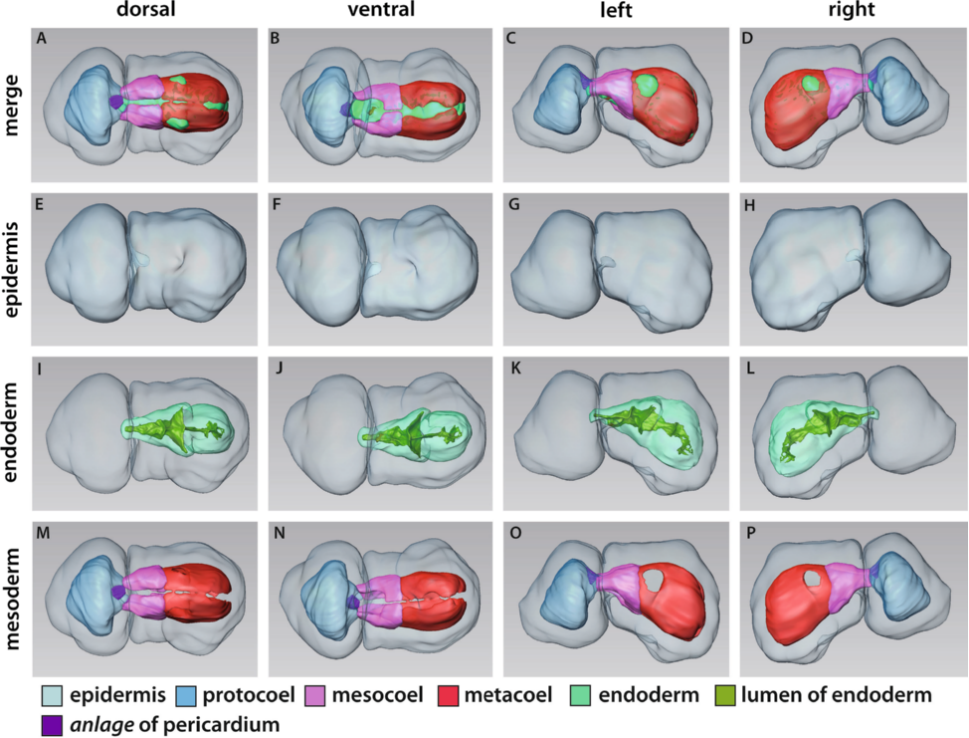
**3D-reconstruction of the embryo of *Saccoglossus kowalevskii *at early kink stage (~ 96 h pf).** Rows from left to right: dorsal, ventral, left and right view. Columns from up to down: The merge row **(A-D)** shows the embryo with all reconstructed structures. Note the asymmetric development of the anlagen of the gill pores (*double arrowheads*) in C and D and O and P. Epidermis row **(E-H)** shows the external shape of embryo. The telotroch is not shown. Endoderm row **(I-L)** reveals the transparent endodermal tissue (*light green*) revealing the subdivision into an anterior pharynx and posterior intestine region. The anlagen of the gill pores (*gpA*) can easily be discerned. The anlage of the stomochord (*stA*) is protruding shortly into the protocoel. Mesoderm row **(M-P)** shows the position of the anterior protocoel (*blue*) and the paired meso- (*pink*) and metacoelic (*red*) compartments. The pericardium (*purple*) is bulb-shaped and protruding into the protocoelic cavity. Download interactive 3D-PDF as Additional file 5. *co* collar, *mo* mouth opening, *stA* anlage of the stomochord, *tr* trunk.

**Figure 7:**
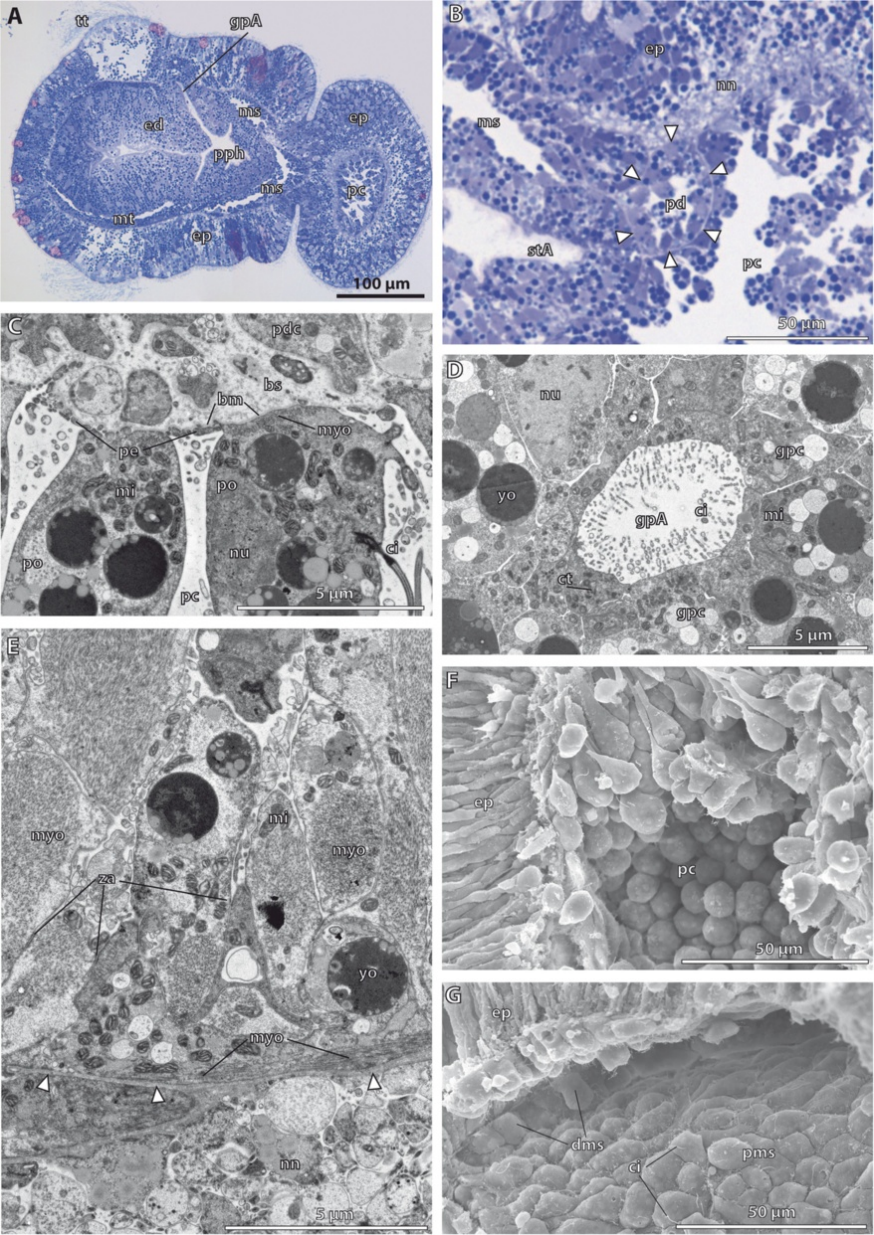
**Histology and fine structure of the late kink stage in *Saccoglossus kowalevskii *(~ 96 h pf). (A)** sagittal section showing the internal organization. The anlagen of the 1st gill pores (*gpA*) are visible. The meso- (*ms*) and metacoelic (*mt*) cavities enlarge to form central lumina. **(B)** The pericardium (*pd*) is surrounded by a basement membrane (*arrowheads*) and furthermore bulb-shaped by protruding into the protocoelic cavity (*pc*). **(C)** The protocoelic cells lining the pericardium are differentiated into podocytes (*po*) resting on prominent blood sinus (*bs*) nested underneath the basement membrane (*bm*). **(D)** The elliptic anlage of the gill pore is constituted by monociliated cells though, the cytoplasm of individual cells contain several centrioles (*ct*) indicating ciliogenesis. **(E)** The majority of the protocoelic cells are differentiated into myoepithelial cells. Inner longitudinal muscle cells alternate with circular muscle cells as indicated by the arrangement of myofilaments (*myo*). **(F)** SEM of a dissected embryo revealing the goblet-shape of the apical cell processes of the protocoelic cells. **(G)** The meso- and metacoelic lining cells constitute a sqamous epithelium holding a single cilium (*ci*) at the cell surface. *dms* distal mesodermal cell, *ed* endoderm, *ep* epidermis, *gpc* gill pore cell, *mi* mitochondrion, *nn* nerve net, *nu* nucleus, *pdc* pericardial cell, *pe* pedicels, *pms* proximal mesodermal cell, *pph* primordal pharynx, *stA* anlage of the stomochord, *yo* yolk, *za* zonula adherens.

The embryos at 96 h pf measure about 470 μm in total length and can be divided into three body regions, the anterior proboscis region, the middle collar region, and the kinked trunk region (Figures 6A-H and 7A, Additional file 5). The proboscis is slightly elongated and tapers at the anterior end. It measures about 140 μm in length and is separated from the collar region by a circular constriction.

The endoderm is surrounded by the paired collar and trunk coeloms. The endoderm is connected to the exterior by means of a mouth opening (Figure 6B,C). The position of the mouth opening shifted from ventral to anteroventral and is now located deeply within the circular constriction. Although the lumen of the endoderm is still compressed at this stage, specific regions of the prospective digestive tract can be distinguished due to conspicuous constrictions of the endodermal wall. Within the buccal cavity, a short diverticulum is protruding anterodorsally into the protocoel measuring about 40 μm in total length (Figures 6K,L and 7B). This diverticulum constitutes the anlage of the stomochord and possesses a central narrow lumen that is continuous with the lumen of the buccal cavity. The buccal cavity is continuing into the pharynx region posteriorly. At the late kink stage, the anlagen of the first pair of gill pores are distinguishable as tubular dorsolateral outgrowths of the pharyngeal wall (Figures 6I,J and 7A). These outgrowths are still blindly closed at their distal ends, where the endodermal tissue is directly adjoining the ectodermal layer. Careful analysis of the complete serial sections and the 3D-reconstruction reveal that the right and left anlage of the gill pores are slightly asymmetrical. The areas of attachment of endoderm and epidermis are circular on both sides, but the area on the left side is bigger (Figure 6C,D,O,P). The pharyngeal region ends after about 120 μm by a slight annular constriction just posterior to the anlagen of the gill pores and continues into the intestine. The intestine is the sac-like posterior part of the digestive tract, situated in the kinked trunk region of the embryo. The intestinal lining is tall, leaving a flattened narrow lumen (Figure 7A). The intestine is not subdivided further and ends blindly as the future anus is not opened yet.

The shape of the protocoel follows that of the external appearance of the proboscis. It tapers anteriorly and is extended posteriorly to border the paired mesocoelic cavities (Figure 6A-D, M-P). The pericardial sac is suspended dorsally in the posteriormost region of the protocoel. It is pear-shaped measuring 25 μm in width and 30 μm in height and projects into the protocoelic cavity (Figure 7B). The pericardial sac is attached dorsally to the epidermis and adjoined ventrally by the stomochord.

The paired mesocoela surround the anterior digestive tract completely omitting only the ventral mouth area (Figure 6A-D,M-P). The left and right mesocoela adjoin in the ventral and dorsal midline thereby forming longitudinal mesenteries. Moreover, each of the mesocoela is extended anteriorly by sending lateral extensions into the basis of the proboscis (Figure 6O,P). The trunk region is occupied by the paired metacoelic cavities ensheathing the gill region and intestine from the left and right side (Figure 6A-D,M-P). The left and right coeloms adjoin each other in the ventral and dorsal midline, but stay separated by mesenteries.

The main changes compared to the early kink stage concern the ongoing development of the pericardial complex and the anlagen of the gill pores. The following description of the fine structure will therefore particularly focus on these changes.

#### Fine structure of the endoderm

All endodermal cells are still comparably undifferentiated and constitute a pseudostratified monociliated epithelium similar to the one found in the early kink stage. Of interest are the cells lining the lateral outgrowths of the pharyngeal wall, namely the anlagen of the gill pores. These ducts of the gill pores are tubular and elliptic in cross sections. The cells lining the still blind terminal endings of the ducts are monociliated cells, although the cytoplasms of individual cells contain several centrioles indicating ciliogenesis.

#### Fine structure of the protocoel

The cells lining the protocoel can be divided into two different types of monociliated epithelial cells according to differences in form and function. The first type of cells is lining the pericardial sac and is differentiated into podocytes (Figure 7C). These bulbed-shaped cells may be slightly elongated protruding about 7 μm into the protocoelic cavity. The cytoplasm contains several vesicles of yolk and a central nucleus, somewhat irregular of shape. Moreover, most of the abundant mitochondria are placed closely to the basal myofilaments (Figure 7C). Such myofilaments are also well developed within the fine pedicels arising from the podocytes. The majority of myofilaments are longitudinally arranged. The podocytes rest on a basement membrane beneath which a prominent haemal sinus is present (for more details see [15]).

The second type of epithelial cells comprises well developed myoepithelial cells that are interconnected by apical adherens junctions (Figure 7E). They form either a distinct outer circular muscle layer or individual longitudinal muscle strands spanning the lateral and ventral portions of the protocoel. The centre of the protocoel on the other hand is occupied by the fluid-filled coelomic cavity. The basal portion of the circular muscle cells are flattened and elongated in circular direction and attached to the basement membrane via hemidesmosomes. Several mitochondria positioned next to the basal circular myofilaments are typically found in this portion. The cytoplasm is of medium electron density in electron micrographs. The basal portion of these cells is connected to a goblet-shaped perikaryon via a slender elongated neck region (Figure 7E,F). The perikaryon projects deeply into the protocoelic cavity and contains vesicles of yolk. Furthermore, organelles such as rough ER and a golgi apparatus are present. The latter is located close to the basal structures of the single cilium that emerges from the cell surface. The longitudinal muscle cells to the contrary show only limited connection areas to the basement membrane and are located interspersed between the circular muscle cells (Figure 7E). Some cells span the protocoelic cavity laterally and are connected to a basement membrane on both ends, whereas others protrude into the cavity with a goblet-shaped soma (Figure 7F). The cytoplasm appears electron light and is primarily filled with myofilaments running in longitudinal direction. Numerous mitochondria are commonly found accompanying the myofilaments. Other organelles such as the rER, nucleus or vesicles of yolk are restricted to the areas without myofilaments, namely the perikaryon or the basal portions.

#### Fine structure of the mesocoel and metacoel

As stated above, the mesocoelic and metacoelic cavities do not show further differentiations in their ultrastructure, compared to the early kink stage. Both are secondary body cavities lined completely by monociliated squamous epithelia (Figure 7G). The cells rest on a basal lamina and the cytoplasm is filled with numerous yolk granules. Most of the cells are elongated in anterior to posterior direction, measuring between 8 μm and 16 μm in length.

### 1 gill slit stage

#### Gross morphology

**Figure 8:**
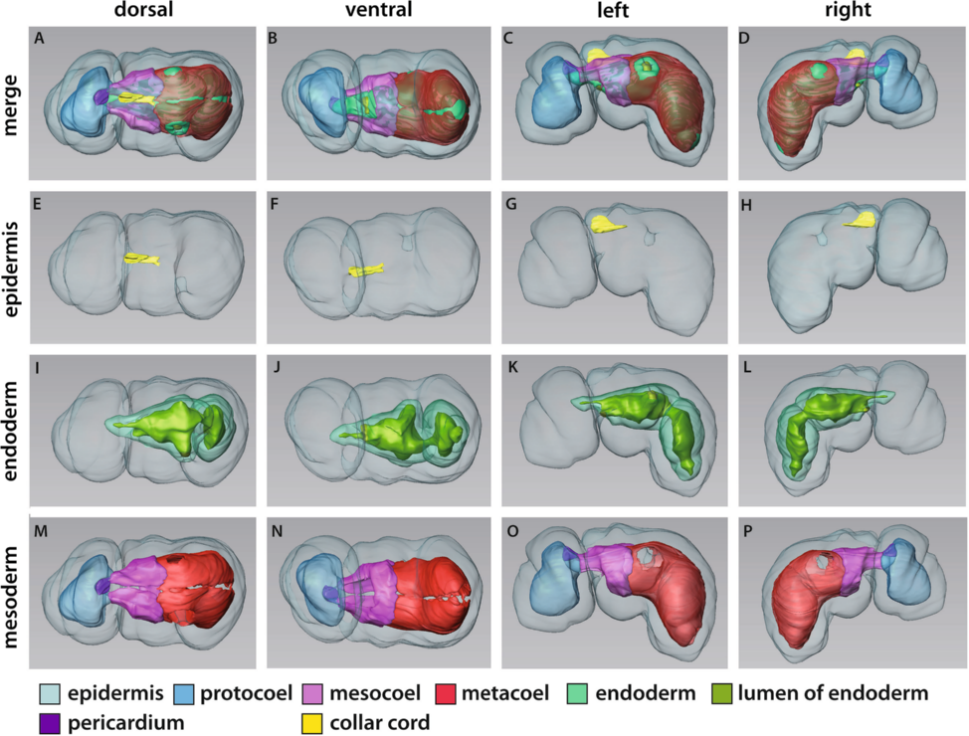
**3D-reconstruction of the embryo of *Saccoglossus kowalevskii *at 1 gill slit stage (~ 132 h pf).** Rows from left to right: dorsal, ventral, left and right view. Columns from up to down: The merge row **(A-D)** shows the embryo with all reconstructed structures. The first gill pore (*gp*) is opened on the left side whereas the right side is still closed. Epidermis row **(E-H)** shows the external shape of embryo. The telotroch is not shown. The collar cord (*cc*) is invaginated by a process similar to chordate neurulation. Endoderm row **(I-L)** shows the transparent endodermal tissue (*light green*) revealing the right-curved course of the oesophagus (*oe*). Only the left gill pore is opened. The still short stomochord (*st*) is protruding into the protocoel. At the posterior end of the digestive tract a hindgut region (*hg*) can be distinguished. Mesoderm row **(M-P)** shows the position of the anterior protocoel (*blue*) and the paired meso- (*pink*) and metacoelic (*red*) compartments. The pericardium (*purple*) is protruding into the protocoelic cavity. Download interactive 3D-PDF as Additional file 6. *i* intestine, *mo* mouth opening, *ph* pharynx.

The embryos of this stage are about 510 μm long. The dorsal epidermis of the collar region shows a longitudinal depression, the neural groove where the neurulation of the collar cord proceeds (Figures 8A-H, Additional file 6). The posterior part of the collar cord is already invaginated underneath the epidermis and forms a hollow tube, whereas the anterior part is still an open groove. At this developmental stage the 1st pair of gill pores is present just behind the collar region. The pores open asynchronously, namely first on the left side (Figure 8A,C, D). The trunk region posterior to the gill pores is kinked and elongated ventrally to an extend of 140 μm.

**Figure 9:**
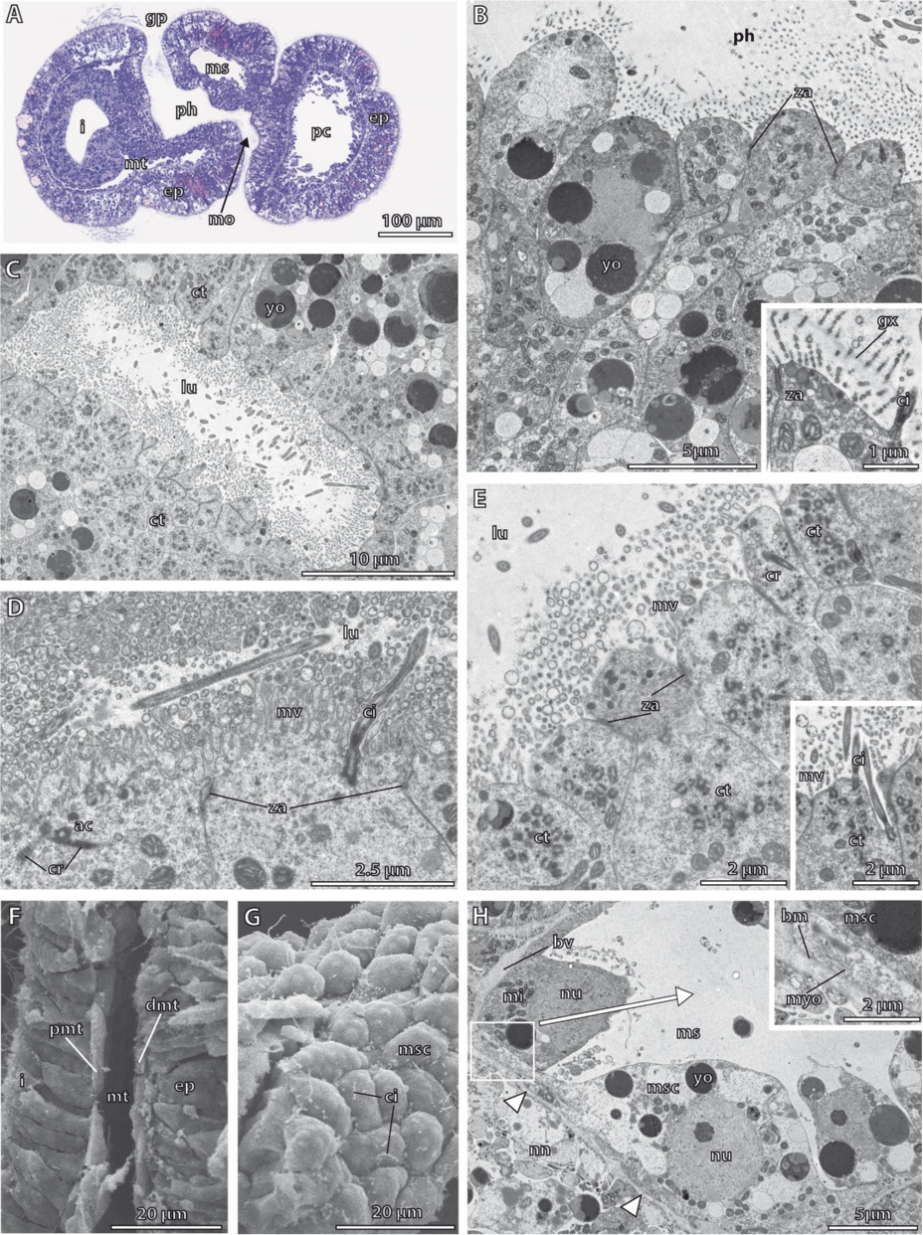
**Histology and fine structure of the 1 gill slit stage of *Saccoglossus kowalevskii *(~ 132 h pf). (A)** Slightly oblique sagittal section displaying the opening of the mouth (*mo*) and the left gill pore (*gp*). **(B)** Pharyngeal lining cells. Inset: The slender microvilli (*mv*) produce a glycokalyx (*gx*) covering the cells. **(C)** Duct of the gill pore. Cilia (*ci*) and microvilli are projecting into the lumen of the duct. **(D)** High mag of the apical cell surfaces of the cells lining the intestinal region. The entire surface area is enlarged to form microvilli. E The cells lining the duct of the gill pores are interconnected by zonulae adherentes (*za*). The apical cytoplasm contains numerous centrioles (*ct*). Inset: The duct lining cells are developing into multicilated cells. **(F)** The proximal (*pmt*) and distal (*dmt*) cells of the metacoel constitute flattened epithelial cells. **(G)** Epithelial cells constituting the mesocoelic lining. **(H)** Cross section of the mesocoelic lining cells (*msc*). Blood vessels (*bv*) are situated between the basement membranes (*arrowheads, bm*). Inset: The mesocoelic cells contain myofilaments (*myo*) within the very basal portions of the cytoplasm. P *ac* accessory centriole, *cr* ciliary rootlet, *ep* epidermis, *i* intestine, *lu* lumen, mi mitochondrion, *ms* mesocoel, *mt* metacoel, *nn* nerve net, *nu* nucleus, *pc* protocoel, *ph* pharynx, *yo* yolk.

The endoderm comprises the digestive tract and is subdivided into five different regions. The developing stomochord measures about 80 μm in length and projects anteriorly into the protocoel (Figure 8K,L, Additional file 7: Figure S7C,D). Its central lumen is continuous with that of the buccal cavity. The buccal cavity lies within the collar region and opens to the exterior through the mouth on the ventral side (Figures 8K,L and 9A). The entire lumen of the digestive tract is enlarged and dilated from the time the 1st pair of gill pores opened. The buccal cavity continues into the pharynx region that is situated within the anterior trunk region, just behind the collar. Here, one pair of lateral outgrowths are developed to form the 1st pair of gill pores. The endodermal tissue is directly connected to the epidermis and constitutes an opened circular-shaped gill pore on the left side only. The corresponding right side is still closed (Figure 8C, D). The pharynx region ends shortly behind the gills and continues into the intestine posteriorly. This area is discernible by an annular constriction (Figures 8I-L and 9A). Pharynx and intestine are joined by the oesophagus, a short and small tubular connection that is running in the right half of the body in relation to the dorsal midline. The intestine is a somewhat sac-like structure and about 130 μm long. The prospective hindgut region can be distinguished by a shallow circular constriction about 60 μm from its posterior blind ending. The proboscis is occupied by the protocoel and is lined by a muscular epithelium composing the proboscis musculature (Figure 9A). The pericardial coelom is suspended dorsally in the posteriormost region above the stomochord and measures about 32 μm in width and breadth (Additional file 7: Figure S7F). It is attached posterodorsally to the epidermis and projects into the protocoel anteriorly together with the adjoining stomochord.

The paired mesocoel fills the collar region (Figures 8A-D, M-P and 9A). The left and right mesocoela encircle the buccal cavity and are bordered anteriorly by the protocoel and posteriorly by the metacoel by means of vertical septa. Both coelomic cavities are separated in the dorsal and ventral midline by longitudinal mesenteries. The metacoel fills the trunk region completely. It is a paired structure as well and ensheaths the pharynx by surrounding the gill pores, the intestine, and the hindgut regions (Figures 8A-D,M-P and 9A). All coelomic cavities at this stage are sac-like structures with no coelomopores present.

#### Fine structure of the endoderm

The entire digestive tract is composed of a pseudostratified, monociliated epithelium that is covered by numerous short and slender (2 μm length, 60 nm diameter) microvilli at the apical cell surfaces (Figure 9B and inset, Additional file 7: Figure S7D). Albeit the enormous enlargement of the lumen of the endoderm compared to the late kink stage, the endodermal cells are still comparably undifferentiated and no specialised cell types as e.g. glandular cells are found. However, the organization of the apical cell surface, predominantly the ciliation, differs in certain endodermal regions and will be described in more detail in the following.

Between the monociliated endodermal cells and close to the mouth opening at the ventrolateral side, few individual multiciliated cells are interspersed containing a very electron light cytoplasm compared with the neighbouring cells (Additional file 7: Figure S7E). These cells contain several basal complexes in accordance with the increased number of cilia. As mentioned above, the anlage of the stomochord is visible as a rod-like extension of the anterior epithelium lining the buccal cavity (Additional file 7: Figure S7C,D). It is completely surrounded by extracellular matrix and appears oval in cross section (Additional file 7: Figure S7A,B). The epithelial cells are equipped with one cilium and several microvilli at the apical cell surface. Furthermore, neighbouring cells are interconnected by adherens junctions. However, the cells comprising the anlage of the stomochord are not further differentiated.

In the posterior pharynx region the 1st pair of gill pores is developed (Figure 8A,B, and 9A). The duct region leading to the gill pore opening is almost slit-like measuring 25 μm in length and 8 μm in breadth (Figure 9C, S3C). The lining cells contain a conspicuously high number of centrioles within the cytoplasm of the cells (Figure 9C,E and inset). Furthermore, numerous mitochondria are placed aside the centrioles within the cytoplasm. The increased number of centrioles together with associated basal structures is a clear indication for continued immense ciliogenesis in these cells as the cells will develop into heavily ciliated cells.

The intestine region is composed of columnar and monociliated cells, whose microvilli differ from those present on the cells lining the buccal cavity and pharyngeal region. Here, the entire apical cell surface is enlarged to form 1.5 μm short, but 160 nm thick microvilli (Figure 9D). The cilia are anchored to the cytoplasm by two striated rootlets. The cells contain the common set of organelles and several granules of yolk placed basally within the cells. Adjacent cells are interconnected by apical adherens junctions (Figure 9D).

#### Fine structure of the protocoel

The cells that constitute the outer circular and inner longitudinal muscle layer show the same ultrastructural features as the myoepithelial cells described for the late kink stage. We could not detect any further differentiations or changes in the shape or arrangement of the cells and will therefore focus on the changes occurring in the development of the proboscis pore and the podocytes. On the left side of the posteriormost region a short tubular outgrowth of the protocoel, the prospective proboscis pore duct, is visible. The duct is not connected to the exterior yet, and the cells lining this duct are comparably undifferentiated. In contrast to the myoepithelial cells forming the proboscis musculature, the cells lining the duct do not contain substantial bundles of myofilaments (Additional file 7: Figure S7G).

The protocoelic cells lining the pericardial sac from the protocoelic side rest on a basement membrane which covers a prominent blood vessel situated between basement membranes (Additional file 7: Figure S7F,H). There, the protocoelic cells have developed into podocytes, while still containing numerous yolk granules. The apical surfaces of the podocyte cells bear a single cilium anchored to the cell by a basal body and one striated rootlet fibre. The pedicels of neighbouring podocytes form fenestrations between them (Additional file 7: Figure S7H). Bundles of myofilaments can be found in the basal portions of the podocytes as well as in larger pedicels. The myofilaments are exclusively arranged in transverse direction and therefore correspond to the findings in the late kink stage.

#### Fine structure of the mesocoel and metacoel

Both, mesocoel as well as metacoel are exclusively lined by a squamous and monociliated epithelium (Figure 9F,G). The cells are interconnected by adherens junctions and rest on a basal lamina (Figure 9H). The area containing the nucleus usually bulges into the coelomic cavity. Furthermore, the cells taper laterally and send flattened elongations to their neighbouring cells. Myofilaments are present within the basal portion of the epithelial lining cells (Figure 9H inset) and are more distinct in areas where a blood vessel is present within the *ecm* these cells rest on. Obviously, the myofilaments are supposed to perform contractile function for maintaining blood flow.

### 2 gill slit stage

#### Gross morphology

**Figure 10:**
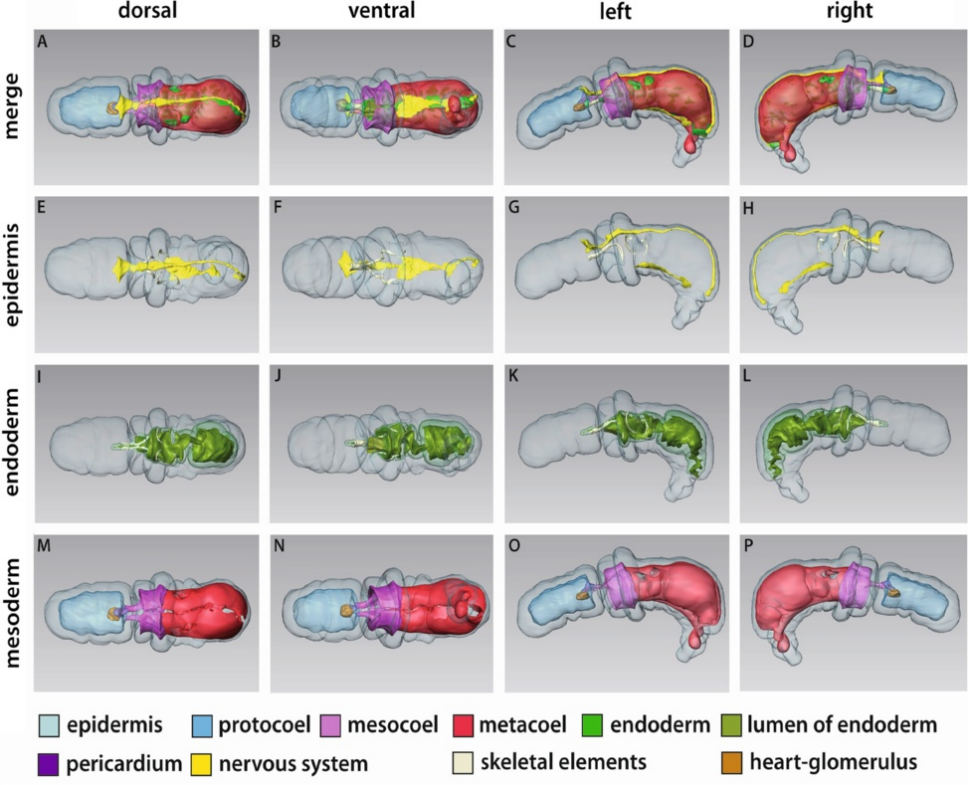
**3D-reconstruction of the juvenile of *Saccoglossus kowalevskii *at 2 gill slit stage (~ 432 h pf).** Rows from left to right: dorsal, ventral, left and right view. Columns from up to down: The merge row **(A-D)** shows the juvenile with all reconstructed structures. Note the characteristic post-anal tail (*pat*). Epidermis row **(E-H)** shows the external shape of juvenile, condensed nervous structures and the skeletal elements. There is only one skeletal rod (*skr*) developed on the right side yet, whereas two are already present on the left side. Endoderm row **(I-L)**: The stomochord (*st*) is projecting into the protocoel and supported ventrally by the proboscis skeleton (*sk*). The oesophagus (*oe*) is positioned asymmetrically on the right body half of the juvenile. The anus (*an*) is opened at the posterior end of the hindgut region (*hg*). Mesoderm row **(M-P)** shows the position of the anterior protocoel (*blue*) and the paired meso- (*pink*) and metacoelic (*red*) compartments. The glomerulus (*gl*) is visible covering the anterior tip of the stomochord. Download interactive 3D-PDF as Additional file 8. *cc* collar cord, *dns* dorsal nerve cord, *gp* gill pore, *i* intestine, *pd* pericardium, *ph* pharynx, *ps* proboscis stem, *vnc* ventral nerve cord.

**Figure 11:**
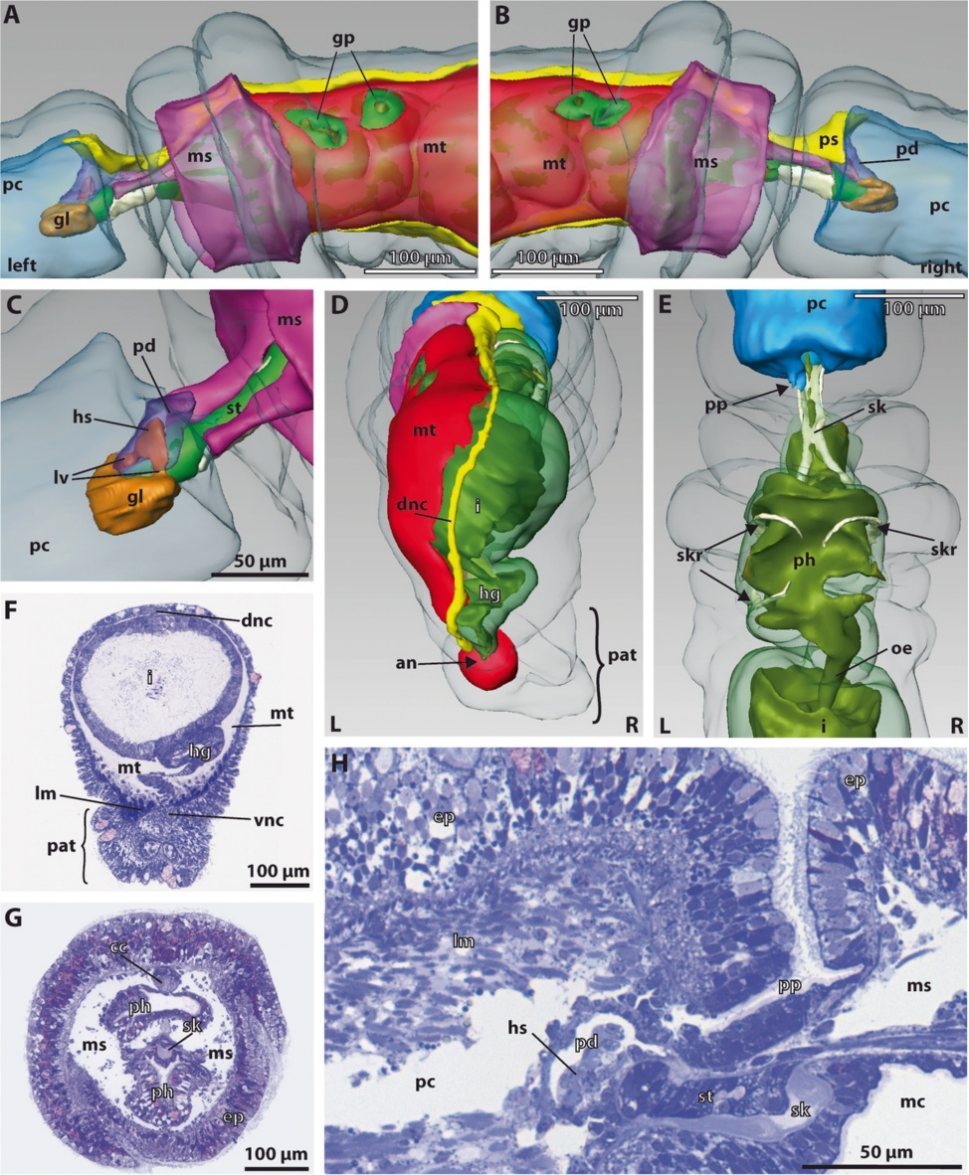
**Detail images of the juvenile of *Saccoglossus kowalevskii *at 2 gill slit stage (~ 432 h pf). (A-E)** 3D-reconstruction, **(F-H)** histological sections. **(A,B)** Detail images showing the left and right sides of the collar and anterior trunk region. Note the asymmetric development of the gill pores (*gp*). **(C)** The pericardium (*pd*) is overlying the heart sinus (*hs*), which is in turn connected to the glomerulus (*gl*) by a pair of lateral vessels (*lv*). Note, the dorsal vessel which supplies the heart sinus from posterior is not shown. **(D)** View from posterior, the right meso- and metacoel are hidden. The dorsal nerve cord (*dnc*) terminates just before the anus (*an*). The metacoel is extended into the post-anal tail (*pat*). **(E)** Dorsal view focussed on the pharyngeal region (*ph*). The skeletal rods (*skr*) develop asymmetrically and ar of unequal number. The proboscis pore (*pp)* opens dorsally on the left side of the base of the proboscis. **(F)** Cross section of the trunk region. The ventrolateral metacoelic cells constitute the substantial longitudinal muscles (lm*)* of the trunk. **(G)** Cross section of the collar region. The collar cord (*cc*) runs within the dorsal mesentery. **(H)** Longitudinal section showing the position of the proboscis pore, stomochord, pericardium and heart sinus. The majority of the protocoelic cavity (*pc*) is filled with longitudinal muscles. Anterior is to the left. *ep* epidermis, *hg* hindgut, *mc* mouth cavity, *ms* mesocoel, *mt* metacoel, *oe* oesophagus, *ps* proboscis stem, *sk* proboscis skeleton, *st* stomochord, *vnc* ventral nerve cord.

**Figure 12:**
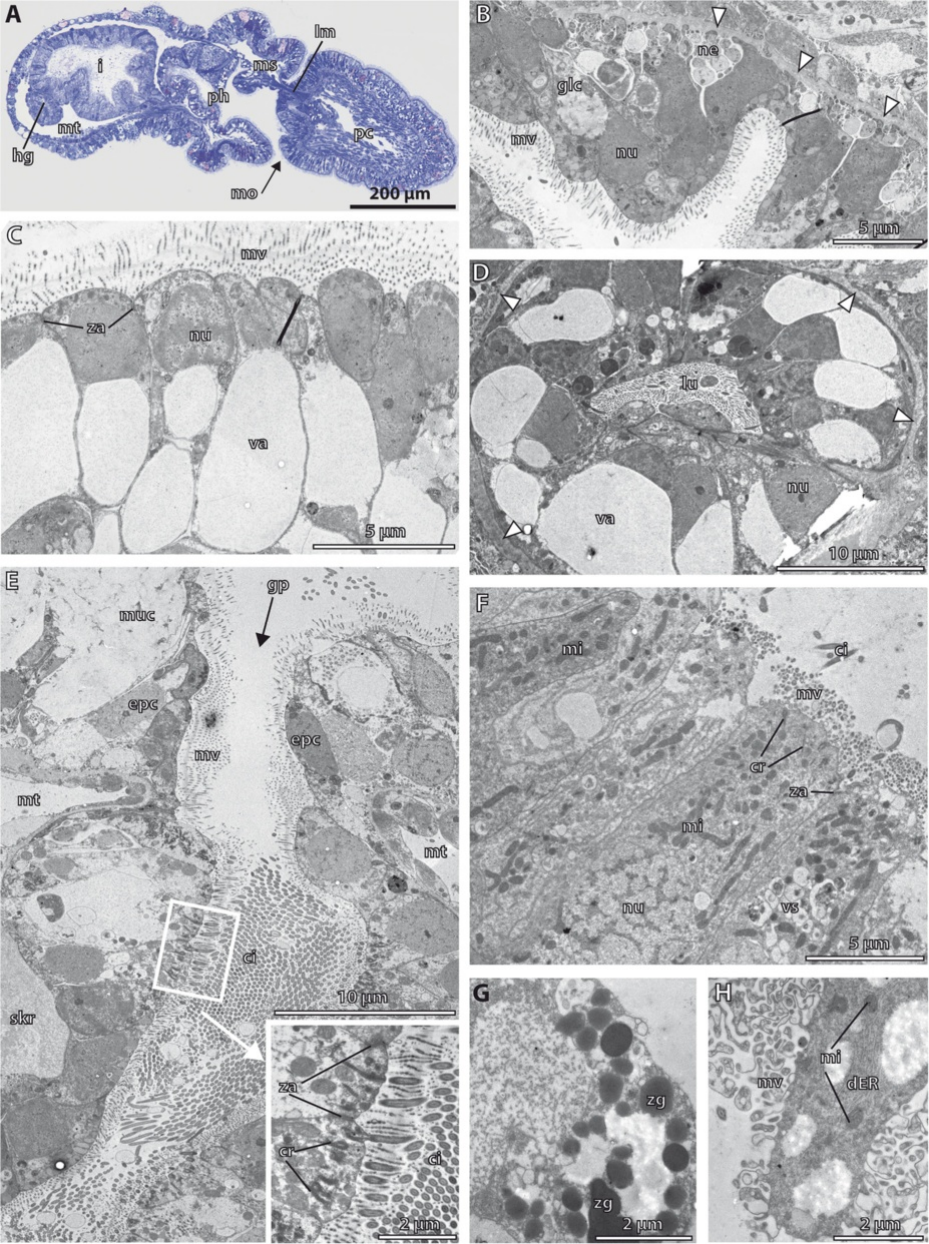
**Histology and fine structure of the juvenile of*Saccoglossus kowalevskii*at the 2 gill slit stage (~ 432 h pf). (A)** Histological section, **(B-H)** Transmission electron micrographs. **(A)** Sagittal section showing internal structures. The proboscis stalk is flanked by elongations of the mesocoelic cavity, which are filled with longitudinal muscle strands (*lm*) completely. **(B)** Cells lining the roof of the mouth cavity. Glandular mucus cells (*glc*) are interspersed. **(C)** Vacuolated cells lining the ventrolateral regions of the anterior pharynx. **(D)** The stomochord is oval in cross sections and its vacuolated cells surround a central lumen (*lu*). **(E)** Horizontal section of the gill pore (*gp*). The proximal duct of the gill pore is extremely high ciliated. Inset: high mag of the cilia (*ci*) and their associated basal structures. **(F)** The intestinal lining is composed of columnar cells. **(G**,**H)** special intestinal cells containing zymogen-like granules (*zg*) or distended amount of rough endoplasmatic reticulum (*dER*). *cr* ciliary rootlet, *epc* epidermal cell, *hg* hindgut, *mo* mouth opening, *mi* mitochondrion, *ms* mesocoel, *mt* metacoel, *muc* mucus cell, *mv* microvilli, *ne* neurites, *nu* nucleus, *pc* protocoel, ph pharynx, *skr* skeletal rod, *va* vacuole, *vs* vesicles, *za* zonula adherens.

These juveniles are freely crawling over the substratum and their overall appearance resembles those of the adults in many aspects, except for the reduced number of gill pores, the development of the gonads, and the presence of a post-anal tail (Figures 10A-D, 11A,B and 12A). The small acorn worm is about 800 μm long. The body is separated into a proboscis measuring 330 μm in length that is followed by two epidermal rings demarcating the collar region connecting to a wormlike trunk. The proboscis is connected to the collar region by a narrow, dorsal stalk. At the anterior trunk region two pairs of gill pores are visible dorsolaterally (Figure 11A,B). A post-anal tail, 180 μm long, is developed ventrally at the posterior end of the trunk (Figure 10C,D). This post-anal tail is a unique character of the juvenile stage of harrimaniid enteropneusts and will be reduced later during development. It is a creeping organ without detailed morphological similarities to the chordate tail. The condensed portions of the nervous system, a dorsal and a ventral cord, are developed as two basiepidermal, longitudinal neurite bundles (Figures 10E-H and 11D,F). A ubiquitous nerve net is present basiepidermally, but not shown in the 3D-reconstructions. The anterior part of the dorsal neurite bundle is broadened to form the so-called proboscis stem at the base of the proboscis (Figure 11B,H). The dorsal cord runs in the dorsal midline all the way posterior, terminating just prior to the anus opening at the trunk region (Figure 11D). The dorsal cord is internalized within the collar region to form the collar cord. The ventral cord runs longitudinally at the ventral midline within the trunk region, without extending into the post-anal tail and is also absent from the collar region (Figure 10G,H). The ventral cord is more extensive compared to the dorsal cord and accompanies the substantial ventral trunk musculature that is formed by the myoepithelial lining cells of the paired metacoela (Figure 11F).

**Figure 13:**
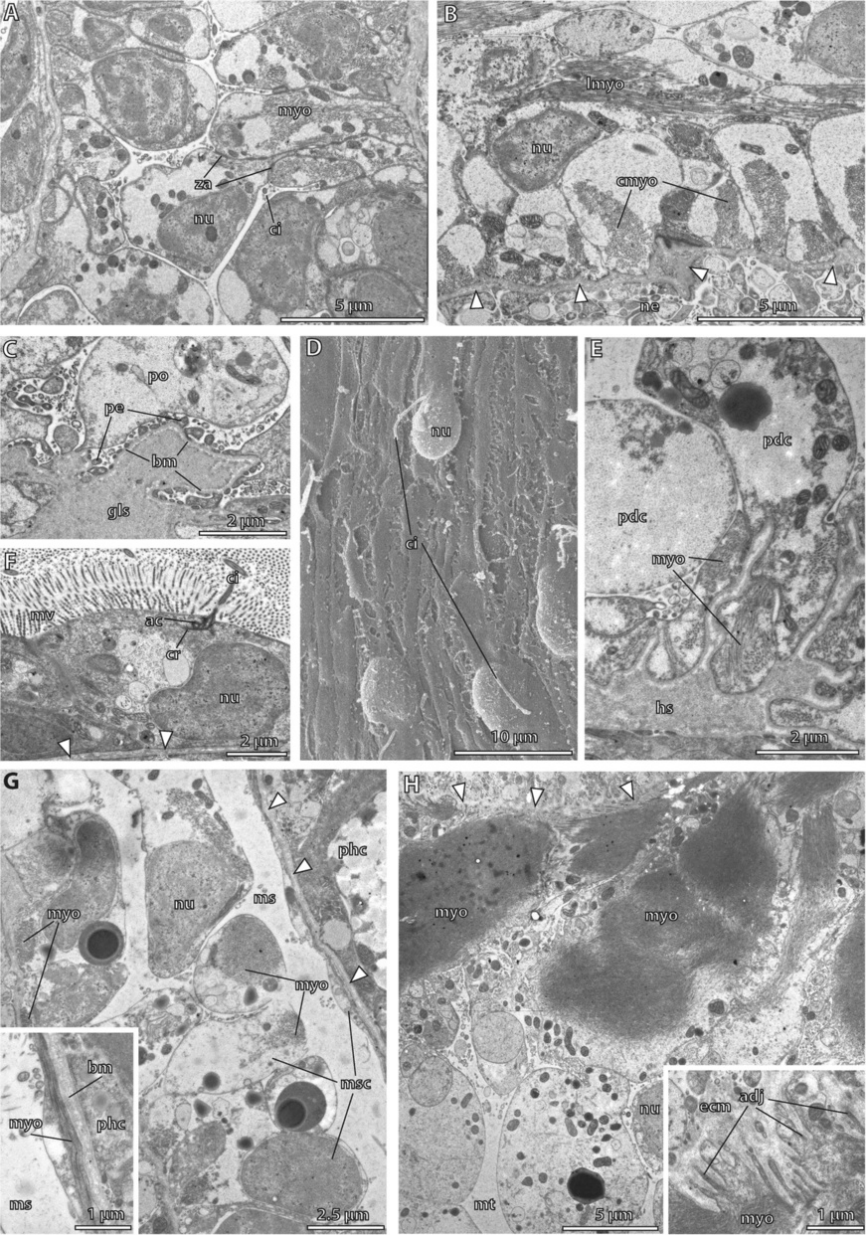
**Fine structure of the juvenile of *Saccoglossus kowalevskii *at 2 gill slit stage (~ 432 hpf). (A)** Cells lining the proximal duct of the proboscis pore. These cells have developed circular myofilaments (*myo*). **(B)** Longitudinal section showing the musculature of the protocoel. Outer circular muscles cells alternate with inner longitudinal muscle cells. **(C)** Podocytes (*po*) resting on the basement membrane (*bm*) covering the glomerular sinus (*gls*). Pedicels (*pe*) form fenestrations between them. **(D)** SEM showing the highly flattened metacoelic cells resting on the intestinal region. The area containing the nucleus is protruding into the coelomic cavity. **(E)** Cross section of the pericardial cells (*pdc*) covering the heart sinus (*hs*). Basal myofilaments are orientated in longitudinal direction. **(F)** Cells lining the distal duct of the proboscis pore. These cells are flattened and highly microvillar (*mv*). **(G)** Cross section of the mesocoelic lining cells (*msc*). Myofilaments of the somatic lining are longitudinally arranged. Inset: in contrast, myofilaments of the visceral lining cells are orientated in circular direction. **(H)** The somatic lining of the metacoel is hyperdeveloped to form the substantial longitudinal strands of the ventral musculature within the trunk region. Inset: numerous adhesion plaques (adj) anchor the myofilaments to the extracellular matrix (*ecm*). *ac* accessory centriole, *ci* cilium, *cr* ciliary rootlet, *cmyo* circular myofilaments, *lmyo* longitudinal myofilaments, *ms* mesocoel, *mt* metacoel, *ne* neurites, *nu* nucleus, *phc* pharyngeal cell, *za* zonula adherens.

The proboscis is occupied by the protocoel that is almost completely filled by circular and longitudinal muscle strands (Figures 10M-P, 11H, 12A and 13B). The protocoel is connected to the exterior by a small proboscis pore, which is located posterodorsal on the left side at the base of the proboscis (Figure 11E,H). This coelomopore is part of the metanephridial excretory system together with the heart-kidney complex that is suspended medially in the posteriormost region of the protocoel (Figures 10M-P, 11C,H and 12A). The heart-kidney is composed of the pericardium, the supportive stomochord and the glomerulus that spans the anterior tip of the pericardium and stomochord (Figure 11C). The pericardium forms a tubular sac measuring 60 μm in length and 15 μm in breadth. It is placed dorsally above the stomochord and encloses a dilated blood space, the heart sinus, which is located within the *ecm* between the pericardium and stomochord (Figures 11H and 13E). The heart sinus bifurcates anteriorly into two anteriolateral vessels connecting to the glomerulus (Figure 11F). The glomerulus extends posteriorly to cover the lateral and ventral areas of the stomochord as well. It is about 30 μm long and 35 μm wide and corresponds to the filtrational part of the excretory system.

The stomochord is a rod-like protrusion emerging from the roof of the buccal cavity (Figures 10I-L and 11C), and its vacuolated cells surround a central lumen and their function is supporting the heart-glomerulus complex. Within the proboscis stalk, the stomochord is reinforced ventrally by the proboscis skeleton. The y-shaped skeleton bifurcates posteriorly when entering the collar by sending one lateroventrally curved branch to each side of the buccal cavity (Figure 10E-L). Further skeletal elements comprise rods of the gill bars within the pharyngeal region (Figure 10G,H,K,L). The skeletal rods are crescent-shaped and taper at both ends, where they are continuous with the *ecm* of the pharynx. Three skeletal rods are discernable at this stage of development, two on the left side whereas only one is found on the right side (Figure 11E). This uneven number of gill bars corresponds to the asymmetric development of the gill pores already observed in earlier stages. Furthermore, the two gill pores on the right side are not separated yet by means of a mesodermal gill bar which will then develop later together with the skeletal rod (Figure 11A, B). In fact, the gill pores are developed as simple tubular ducts with no further subdivision. The pharyngeal region containing the gill pores is about 180 μm long and enlarges anteriorly into the buccal cavity where the anteroventral mouth is located (Figure 12A). The pharynx is connected posteriorly to the intestine by means of a 70 μm short and right-curved oesophagus (Figures 10I,J and 11E). The oesophagus is barely 20 μm in diameter, but opens into the considerably wider lumen of the intestine. The intestine is 190 μm long and kinked ventrally at the transition to the hindgut. This transition can be recognized by a shallow circular constriction (Figures 10I-L and 11D,F). The hindgut region ends after 100 μm and terminates into an anus opening dorsally in respect to the post-anal tail.

The paired mesocoelic cavities occupy the collar region (Figures 10M-P and 11A,B). Furthermore, two elongations projecting anteriorly flank the stomochord laterally about 60 μm into the protocoel (Figure 11C). These elongations are filled with longitudinal muscle strands (Figure 12A), which obviously support the thin proboscis stalk and move the entire proboscis. The remaining coelomic cavity is fluid-filled and lined by flattened epithelial cells. Left and right coeloms are separated bilaterally by a mesentery by which the collar cord is surrounded as well (11G). At the posterior end of the collar region the coelomic cavities are blindly closed and bordered by the posteriorly metacoela by means of vertical septa. There is no coelomoduct or coelomopore developed yet, as described for the adult acorn worm [54]. The paired metacoela surround the pharyngeal region, intestine and hindgut completely and continue ventrally with sac-like elongations into the post-anal tail (Figures 10M-P and 11D,F). The metacoela are pierced by the pharyngeal tissue that forms the gill pores at the anterior region of the trunk (Figure 11A,B). The metacoelic cavities are lined by two different types of cells. The dorsal and dorsolateral portions are occupied by flattened epithelial cells, whereas the ventral and ventrolateral areas are lined by the aforementioned substantial longitudinal muscle strands (Figure 11F). Left and right metacoela are separated by mesenteries that keep the digestive tract in place.

#### Fine structure of the endoderm

The anterior pharynx region, the buccal cavity is constituted of different epithelial cell types. The lateral and ventral portions are lined almost exclusively by highly vacuolated cells (Figure 12C). The single, clear and large vacuole may be of hydrostatic function to stabilize the shape of the mouth cavity. The nucleus is either displaced to the very basal part within the cytoplasm or it is delaminated together with the remaining organelles to the apical cytoplasm, because of the voluminous vacuole. Furthermore, the cells are equipped with one cilium and numerous microvilli at the apical cell surface. The roof of the buccal cavity in contrast, is lined by epithelial cells whose cytoplasm is electron dark is densely packed (Figure 12B). No vacuoles are present in these cells. These non-specialized cells are accompanied by mucous gland cells. The gland cells are filled with electron-lucent secretory product packed in cisternae. Some neurites are present between the basal parts of the cells (Figure 12B).

The stomochord is lined by a monolayered epithelium, which is highly columnar and composed of vacuolated cells (Figure 12D). The cells are interconnected by adherens junctions and have slender microvilli and a single cilium, protruding into the medial lumen. The single, large, membrane-bound vacuole is filled with flocculent precipitate and occupies most of the cytoplasm. The nucleus and other cell organelles are confined to the remaining peripheral cytoplasm.

The proximal region of the duct of the gills is composed of columnar cells which are extremely densely ciliated (Figure 12E and inset). Each cell bears numerous cilia at its apical cell surface of which each is anchored by one vertical rootlet into the cytoplasm (Figure 12E inset). Several mitochondria are always placed close to the basal structures of the cilia. The cell surface is furthermore covered evenly by 1.3 μm short and 45 nm slender microvilli. In contrast, the distal duct region is built up by mostly monociliated cells. Nevertheless, the cell surface is covered by microvilli as well. Following the duct more and more to the exterior, the cells are connected to supporting epidermal cells and different glandular cells (Figure 12E).

The lumen of the intestine is voluminous and lined by a single layer of epithelial cells (Figure 12F). The shape of the cells ranges from more or less cuboid to columnar, measuring about 17 μm in height. The cells are interconnected by apical adherens junctions and anchored basally to a basement membrane. A single cilium is always developed, although cells bearing several cilia are present as well. The nucleus is usually placed basally within the cytoplasm. Furthermore, the cytoplasm contains a high number of mitochondria and lysosomes. Microvilli are covering the apical cell surface evenly.

In addition, another type of intestinal cell can be distinguished, as the cytoplasm contains distended amounts of rough endoplasmatic reticulum, filling almost the entire cell (Figure 12H). Additionally, several electron dark granules, which resemble zymogen granules typically found in pancreatic exocrine cells in vertebrates, are present in some of these cells (Figure 12G). This type of cell is found predominantly in the anterior portion of the intestine close to the opening of the oesophagus.

#### Fine structure of the protocoel

The major part of the protocoelic lining cells is differentiated into myoepithelial cells that build up the proboscis musculature (Figures 11H and 13B). Cells with circularly arranged myofilaments and cells containing longitudinal myofilaments are present (Figure 13B). Longitudinal muscle cells invade and span the coelomic space to attach to the basement membrane of the opposite wall. The circular muscle cells in contrast, form an outer layer of muscles. The majority of the cytoplasm of the cells is filled with myofilaments.

The duct of the proboscis pore is constituted of a proximal and a distal duct region, lined by different cell types, respectively. The proximal duct is composed of monociliated columnar to cuboid cells, which are interconnected by apical junctions (Figure 13A). No microvilli are developed at the surface and the nucleus is positioned centrally. The basal portions of these cells contain considerable amounts of myofilaments, which are arranged circularly surrounding the duct forming a sphincter muscle (Figure 13A). The transition to the cells forming the distal duct region is quite distinctive as these cells posses numerous microvilli (Figure 13F). Moreover, the cells are monociliated and the shape is more or less cuboid or flattened. The nucleus is placed basally and there are no myofilaments present in the cells lining the distal duct region. At the very distal end of the duct the cells are connected to common supportive epidermal cells, which are usually equipped with several cilia at the cell surface. Because myofilaments are restricted to the proximal duct region it is likely that this part of the duct is derived from the coelothelium of the protocoel. In contrast, the distal region of the duct is composed of cells without myofilaments but equipped with microvilli and probably derived from the ectoderm.

The fine structure of the heart-glomerulus complex has been described in detail in Kaul-Strehlow & Stach 2011 [15] and is only summarized here. The pericardial cell which line the heart sinus are monociliated epithelial cells containing thick and thin myofilaments in their basal portions (Figure 13E). These bundles of myofilaments show repetitive striation in longitudinal sections (see [15] Figure 6B). The glomerulus comprises a highly folded and dilated blood space within the matrix and is lined by podocytes at the protocoelic side. Each monociliated podocyte gives rise to numerous pedicels (Figure 13C). The pedicels may contain myofilaments and furthermore form fenestrations between them ranging from 50 nm to 150 nm.

#### Fine structure of the mesocoel

The mesocoelic lining is composed of a monolayered and monociliated myoepithelium. The somatic cell layer, namely the somatopleura is constituted of cells possessing a somewhat irregular shape (Figure 13G). The cells may be up to 8 μm in height and bulge into the coelomic cavity. The cytoplasm appears to be empty except for the nucleus, several mitochondria and myofilaments, which may be positioned either basally or apically within the cytoplasm. However, all myofilaments within the somatic cell layer are orientated longitudinally (Figure 13G). The visceral cell layer, the visceropleura, in contrast, is composed of an extremely flattened myoepithelium, measuring barely 0.6 μm in height (Figure 13G inset). In these cells, the myofilaments are exclusively arranged in circular direction.

#### Fine structure of the metacoel

The cells lining the metacoelic cavity are similarly differentiated, forming a monolayered myoepithelium. On the ventral side, the somatic metacoelic lining forms the substantial longitudinal musculature (Figure 13H). The cells are comparably large containing enormous bundles of myofilaments within the basal part of the cells. The myofilaments are connected to the basement membrane via numerous adhesion plaques (Figure 13H inset). The apical part of the cells is bulb-shaped and protrudes into the coelomic cavity containing the nucleus. Neighbouring cells are interconnected by apical adherens junctions. The visceropleura in the metacoel is also developed as an extremely flattened epithelium (Figure 13D). Only the area containing the nucleus is bulging into the coelomic cavity. Close to the nucleus a cilium is present (Figure 13D).

## Discussion

### Coelom formation

In principle, two main modes of coelom formation can be distinguished in the two major taxa of bilaterian animals: schizocoely in protostomes and enterocoely in deuterostomes (see e.g., [5]). A definition of both terms was given by Lüter [16], who characterized schizocoely as the development of coelomic epithelium from a solid mass of mesodermal cells that *does not* derive from endoderm. In contrast, if coelomic cavities and epithelia *indeed* originate from endoderm, coelom formation is called enterocoely. Note that according to Lüter’s definition of enterocoely, the mesodermal tissue does not have to pinch off as pouches or evaginations ab initio, instead a central lumen can be acquired secondarily [16]. In the discussion afterwards the authors will explicitly follow Lüter’s definitions.

**Figure 14:**
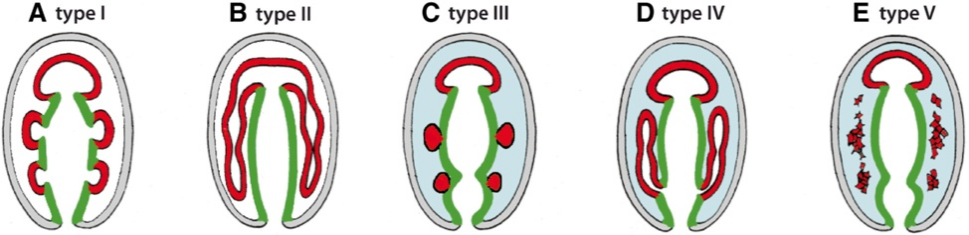
**Coelom formation in Enteropneusta. (A – E)** Schematic drawings of different types of coelom formation compiled and newly colored from text books [3,49,92]. For a more detailed explanation see text and Table 1.

Coelom formation in deuterostomes seems well documented and numerous textbooks have depicted and commented on the diversity of coelom formation especially in enteropneusts (e.g. [1,3,11,49]). Most often this diversity of ontogenetic processes is divided into five different types (see Figure 14) and it is concluded that the variability of coelom formation in a small taxon comprising merely 80 species indicates that this character is of little phylogenetic value. Alternatively, it is possible that the perceived differences in coelom formation in enteropneusts are not as big as they are generally believed to be. Especially, bearing in mind that two of the five types of coelom formation (types I and II below and in Figure 14A and B) have been described from the same genus, i.e. two *Saccoglossus* species. In addition, it is important to realize that the sole criterion to distinguish germ layers accurately is the extracellular matrix (ecm) separating them. The thickness of this ecm, however, is most often well below the power of resolution of light microscopy and can only be documented by transmission electron microscopy in earlier developmental stages [50]. Thus, we re-investigated the ontogenetic coelom formation in *S. kowalevskii* and carefully evaluated the perceived textbook knowledge against the results of this study and the original primary publications.

### Coelom formation: Type I

In type I, all five coelomic compartments – from anterior to posterior: unpaired protocoel, paired mesocoela, and paired metacoela – form as evaginations of the endodermal wall of the archenteron.

The schematic representation of this type of coelom development in enteropneusts roots in the investigations of the early ontogeny of *Saccoglossus kowalevskii* by Bateson [43,44]. Spengel [41] and Morgan [46] doubted the accuracy of Bateson’s study not least because Davis [47] investigated the ontogeny of another *Saccoglossus* species, namely *S. pusillus* and found that the coelom develops differently. This mode of coelom formation is called type II and explained below and in Figure 14B.

### Coelom formation: Type II

In type II, an unpaired anterior sac develops via enterocoely from the archenteron and forms lateral longitudinal extensions. From these lateral extensions the paired mesocoel and metacoel pinches off during later development.

This type of development was described by Davis [47] based on his studies of *S. pusillus*. Because types I and II have been considered to be mutually exclusive [41,46], a re-investigation was needed. Based on our detailed TEM-analysis, we confirm Bateson’s description of type I coelom formation in *S. kowalevskii* and suspect that Davis failed to trace the feeble ecm that separates the coelomic cavities from each other in the beginning. However, although a different development of such closely related species is unlikely, TEM studies on *S. pusillus* will be necessary to settle this dispute.

#### Coelom formation: Type III

In type III, the anterior unpaired protocoel forms as a hollow evagination from the endodermal archenteron, whereas the paired mesocoela and metacoela develop separately from the middle and posterior endodermal archenteron as solid cell masses (Figure 14C).

Morgan [45] described this type of coelom formation in his study of a tornaria larva of unknown species identity. He originally emphasized that, although the paired metacoels seem to be solid, they nevertheless “…are undoubtedly to be regarded as gut pouches or enterocoels,…(p. 414)”. So basically, this type III of coelom formation is similar to type I. Concerning the mesocoela however, the light microscopical resolution has not been sufficient for Morgan to determine, if they arose as epithelial pouches, too ( [45], p. 413).

### Coelom formation: Type IV

In type IV, the anterior unpaired protocoel forms as a hollow evagination from the endodermal archenteron, whereas the paired posterior coeloms develop from postero-lateral evaginations of the archenteron (Figure 14D). These evaginations later on subdivide to form the more anterior mesocoela and the more posterior metacoela.

Type IV of coelom formation was initially suggested by several authors based on their respective studies of tornaria larvae of *Balanoglossus clavigerus*[41,48,55] and *Glandiceps* sp. [56]. The authors themselves however remained cautious especially in respect to the developmental origin of the mesocoela that was either unknown [48,56] or was described rather vaguely [41,55]. Apart from that, type IV of coelom formation has also been described in a recent study on the development of *Balanoglossus misakiensis* from Japan [57]. In this study the authors could convincingly show, that both mesocoela and metacoela indeed originate from the posterior endoderm as a single protrusion that subsequently subdivide.

### Coelom formation: Type V

In Type V, the anterior unpaired protocoel forms as a hollow evagination from the endodermal archenteron, whereas the paired posterior coeloms develop from clusters of mesenchymatic cells in the blastocoel (Figure 14E). These mesenchymatic cells later on multiply and become epithelial surrounding the paired coelomic cavities of the mesocoela and metacoela.

This type of coelom formation was described by Spengel [41] and Morgan [46] in tentaculated tornaria larvae of an unknown ptychoderid species. Both authors lament difficulties in fixation and limited availability of crucial developmental stages. In addition, the origin of the mesenchymatic cells themselves remains unclear.

These different types of coelom formation are contrasted in Table 1, which makes several problems obvious. Most evident is that the common sectioning methods of classical studies combined with limited light microscopical resolution was apparently not adequate to precisely follow the development of the mesoderm in enteropneusts. Added to this, conclusions were sometimes drawn from badly preserved specimens and/or limited availability of important developmental stages. In cases with remaining uncertainties, we recommend a re-investigation using electron microscopy. Urata and Yamaguchi [57] could moreover show that imaging favourable living material with a light microscope of high quality is also a promising method with the additional advantage to avoid fixation artefacts.

**Table 1:** Summary of characteristics of the five types of coelom formation described for enteropneusts according to literature

	**Characteristics accord. to literature**	**Taxon**	**Source**	**Author’s commentary**
Type I (Figure 14A)	Mesocoela & metacoela form as separated, epithelial outpocketings from the middle and posterior gut regions	*S. kowalevskii* (harrimaniidae)	Bateson (1884) [44] present study	Confirmed by ultrastructural investigations (present study)
Type II (Figure 14B)	A single anterior coelomic sac pinches off from the archenteron and subdivides posterolateral into mosocoel & metacoel	*S. puillus* (harrimaniidae)	Davis (1908) [47]	Limited light microscopical resolution. TEM study warranted
Type III (Figure 14C)	Mesocoela and metacoela form from separated, but solid masses of cells from the middle and posterior gut regions	New England Tornaria unknown species (Ptychoderidae*)*	Morgan (1891) [45]	Misenterpretation of the term *solid*, originally described as epithelial enterocoela
Type IV (Figure 14D)	Meso-and mrtacoela originate from a single outpacketing from the posterior gut region that later subdivides	*Glandiceps* sp. (Spengelidae)	Rao (1953) [56]	Origin of mesocoela is not documented in original literature.
		*B.clavigerus* (Ptychoderidae)	Bourne (1889) [55]	Origin of metacoela only described convincingly
			Stiasny (1914) [48]	
			Spengel (1893) [41]	
Type V (Figure 14E)	meso-and metacoela originate from multiple clusters of mesenchymatic cells within the blastocoel	tentaculated Tornaria (Ptychoderidae)	Morgan (1894) [46]	Limited light microscopical resolution. TEM study warranted

For a more detailed explanation see text and Figure 14.

Besides technical improvement, clarification of terminology remains essential. While already a cursory view into the original publications attests that most researchers voiced their statements quite cautiously, these doubts did not enter textbooks establishing seemingly accepted knowledge. Especially the use of the term “solid” referring to type III came to be interpreted as implying a mode of coelom formation different from enterocoely, i.e. schizocoely (e.g., [26]). However, following Lüter [16], enterocoely refers just to the direct origin of mesoderm from endoderm. Thus, type III is probably to be regarded as a form of enterocoely where the coelomic cavity widens between two closely packed, yet epithelial sheets derived from the endodermal archenteron. This may also be the case in coelom formation type V. The problem in this latter case however is slightly different. Here, it is moreover necessary to investigate earlier developmental stages of tentaculated tornaria larvae in order to trace the origin of the mesodermal cells. If they also derive from the archenteron, then type V would be interpreted as a form of enterocoely.

### Comparison to other deuterostomes

Coelom formation in pterobranchs is barely investigated. The only ultrastructural study by Lester [58] shows the elongated larva of *Rhabdopleura normani* with isolated and paired strands of mesocoela and metacoela on both sides of the endoderm and a presumably unpaired protocoel. Lester did not document the origin of the mesoderm, yet the described larva of *R. normani* resembles the early kink stage in *S. kowalevskii.* Thus, a similar origin of the mesocoela and metacoela as in *S. kowalevskii* could be possible. The development in the genus *Cephalodiscus* is poorly known (see summary in [59]).

A comparison of coelom formation with echinoderms is a sight easier as numerous extensive studies are available [17-22]. As introductory mentioned, in many echinoids and also asteroids it has been reported that a single, pouch evaginates from the anterior end of the archenteron, and eventually subdivides into paired axo-, hydro-, and somatocoel (corresponding to pro-, meso-, and metacoel in enteropneusts) on each side (for reviews see [3,23]). This echinoid-type mode of coelom formation is often regarded as being ancestral for Deuterostomia, although differences are reported between echinoderm species [17,26]. For instance, in supposedly basal crinoids, coelom formation is described from separated pockets of the archenteron that derive from the anterior, middle and posterior endodermal regions [60]. Given the difference, that right axocoel and hydrocoel are reduced in crinoids, this description indeed shows similarities to type I of coelom formation present in enteropneusts (see Figure 14A and [3], Figure 57.2).

During the development of amphioxus, multiple pairs of coelomic sacs are formed successively from the endoderm by enterocoely [14,61]. The fate of the coelomic sacs, especially the anterior ones, is much more complex than in ambulacrarian deuterostomes, yet the early larva of amphioxus recapitulates a stage where only three coelomic sacs are developed (see [62], Figure 3), thus highly resembling the condition found in *S. kowalevskii* ( [43,44] and this study). Therefore, similarities between coelom formation in enteropneusts, crinoids as well as cephalochordates are supposed to be inherited from the last common ancestor of ambulacrarians and chordates, and thus may represent the ground pattern of coelom formation in Deuterostomia.

### Conclusions coelom formation

The results from this study and the preceding discussion reveal that coelom formation of type I is present in direct developing harrimaniid enteropneusts such as *Saccoglossus*. The situation in indirect developing enteropneusts (e.g. *Balanoglossus*, *Ptychodera*) however, is much more ambiguous. The only reliable data so far shows that type IV is present in *Balanoglossus* spp [41,48,55,57], whereas re-investigations first have to demonstrate if type III turns out to be similar to type I or where the presumed mesenchymatic cells described in type V in tentaculated tornaria larvae originate from. Nevertheless, it has to be noted that the mesocoela and metacoela never develop by separation from the anterior protocoel. From the available data on coelom formation in enteropneusts alone, is not possible to infer a ground pattern of coelom formation in Enteropneusta, yet an outgroup comparison with the condition in other deuterostomes will help elucidate this issue. The evaluation of coelom formation among other deuterostomes revealed that in basal echinoderms [60], cephalochordates [62] and assumingly also in pterobranchs [58] a single anterior protocoel pinches off of the roof of the archenteron, whereas the paired posterior coeloms independently evaginate from the middle and posterior regions of the endoderm, just as type I described for *Saccoglossus*. Concluding from this, type I of coelom formation represents a plesiomorphic condition for enteropneusts that had been inherited from the last common ancestor of Deuterostomia (LCAD). On the other hand, if indeed type I is assumed to be the ancestral condition of coelom formation in Deuterostomia, then the earlier mentioned echinoid-type of coelom formation has to be regarded as a derived condition that evolved within echinoderms.

### Left-right asymmetries in Saccoglossus

In this study, we generated a surface rendered 3D-reconstruction based on complete serial sections for every developmental stage of *S. kowalevskii*. Our data show that the 1st gill slit opens on the left side, while the corresponding right slit lags behind. Moreover, during the following juvenile stage the temporal asymmetric development is apparent from an unequal number of skeletal gill rods on each side, two on the left, just one developed on the right. Since complete serial sectioning is an accurate, yet time consuming method, a statistical analysis in order to test the significance of our temporal asymmetry is not feasible, due to the small number of specimens processed. Even so, the temporal left-sided asymmetry is present in our investigated specimens, which is why we comparatively discuss them in the light of morphological and molecular genetic data.

Left-right (LR) asymmetry affecting different structures has been acknowledged in almost all deuterostome groups [3,63,64]. For instance, visceral organs, such as liver or stomach in mammals are developed asymmetrically and the heart is generally placed to the left body half [64-67]. In adult ascidians and members of the larvaceans of the taxon Tunicata, the digestive tract is folded asymmetrically [68]. In adult cephalochordates the transition between branchial sac and midgut is shifted to the left-handed side due to the position of the hepatic caecum [61,69,70]. Moreover, quite conspicuous LR asymmetries are present in the larva of cephalochordates during development. The mouth opening penetrates the left larval side while the first row of gill slits successively arises along the ventral midline [69-71] (for reviews see [72,73]). These gill slits correspond to the left gill slits of tunicates and vertebrates and eventually shift to the left body half whereas the right row of gill slits develop later on during metamorphosis [61,73,74]. In most echinoderms, we find almost the entire adult body organized asymmetrically in favour of the left side [18,19,21] (for review see [23]). The bilaterally organized larva of sea urchins and sea stars enters an asymmetric stage during development by forming the rudiment of the juvenile at the left side of the larva [20,22].

In the pterobranch *Rhabdopleura*, the oral lamella, the position of the gonopore, and the gonads in adult animals are described as being asymmetrical by Schepotieff [75]. However, a recent statistical analysis of the position of the gonads done by Sato & Holland [76] could furthermore show that the position of the gonad displays antisymmetry and not directional asymmetry.

In the present study we confirm the asymmetric left-sided position of the proboscis pore in *S. kowalevskii* that has been described by other authors [41,43,49]. It should be mentioned here, that the position of this porus is species dependent and may either be on the left or the right side, and even paired pori exist in some species [11,49,77]. However, a corresponding structure is present in echinoderms in form of the axohydrocoel that opens externally through the madreporic porus or hydroporus in larval echinoderms, respectively [3,23]. Specific similarities between these pori as part of the excretory complex (axial complex in echinoderms and pericard-glomerulus complex in hemichordates) have been intensely studied [3,62,78], and homology of both complexes is generally accepted. However, the homology of the protocoel and porus of echinoderms and hemichordates to chordates is less clear. Based on the anterior position, homology with Hatschek’s left diverticulum or Hatschek’s pit is traditionally suggested [12,79,80], whereas according to similarities in structure, ontogenetic position, and excretory function, homology with Hatschek’s nephridium is emphasized by Stach [62].

Aside from an asymmetric proboscis pore, no further topographical asymmetries have been reported in enteropneusts so far [41,44-48,53,81]. As already stated above, our studied specimens show a slightly temporal asymmetric formation of the gill slits and skeletal gill bars in favour to the left side. In adult enteropneusts, including *S. kowalevskii*, the gill slits are consistently developed bilaterally symmetric [11,29,49,54], thus, the observed asymmetry is only present during development.

Molecular genetic data show that genes such as *nodal*, *lefty* and *pitx* are intimately involved in the establishment of left-right axes in deuterostomes [63,82-84]. In chordate embryos after gastrulation *nodal*, *lefty* and *pitx* are expressed on the left side and are necessary for breaking the primary bilateral symmetry [82]. Interestingly, in sea urchins these genes are indeed required in establishing left-right asymmetries, however, they are expressed mirror-inverted compared to chordates [85]. *Nodal* signalling on the right side inhibits the formation of the rudiment that accordingly develops on the left side of the echinoid larva. In *S. kowalevskii pitx* is expressed in the dorsal midline during development [86], yet *nodal* and *lefty* are expressed on the right side of post-gastrula stage embryos [84], just as in sea urchins. In addition, the opposite expression of *nodal* and *lefty* in ambulacrarians (echinoderms and hemichordates) and chordates further supports the theory of inversion of the D-V axis as proposed by several authors [35,87-89].

### Conclusions concerning asymmetries

Concluding from molecular genetic insights, it is conceivable that the right-sided expression of *nodal* and *lefty* in *S. kowalevskii* results in the left-first development of gill slits. Thus, topological as well as temporal asymmetries corresponding to an asymmetric expression of *nodal* and *lefty* are present in enteropneusts and therefore in all major deuterostome groups. In turn, this fact leads to the suggestion that the molecular toolkit for the establishment of LR-asymmetries has already been present in the LCAD. Of course, further molecular genetic experiments on hemichordates are necessary in order to unravel differences in the *nodal/pitx* regulatory network of echinoderms and enteropneusts to explain the drastic asymmetries present in echinoderms compared to the indeed subtle and only temporal asymmetries in *S. kowalevskii*.

## Materials and methods

Adult specimens of *Saccoglossus kowalevskii* (Agassiz 1873) were collected from intertidal areas near Woods Hole (Massachusetts, USA) in September 2007. Animals were brought to the laboratory, separated according to sex, and kept individually in finger bowls. Animals were kept at 18°C on a seawater-table and inspected frequently. When eggs were spawned they were mixed with active sperm isolated from a ripe male individual and diluted in seawater. These procedures are described in more detail in Lowe [90]. Fertilization envelopes were ruptured using fine forceps and appropriate embryonic stages were collected using Pasteur pipettes. Embryos were relaxed in a mix of 7% MgCl_2_ and sea water (1:1) for 5-10 minutes prior to processing for transmission and scanning electron microscopy (TEM/SEM). The studied embryos are from different inseminations.

For TEM most of the relaxation agent was removed and replace with ice-cold primary fixative containing 2.5% glutaraldehyde in 0.2 M sodium cacodylate buffer (pH 7.2), adjusted to an osmolarity of approximately 800 mosm with the addition of NaCl. Primary fixation was stopped after 45 minutes with three buffer rinses for 10, 15, and 20 minutes. Primary fixation was followed by 30 minutes of postfixation with 2% OsO_4_ in sodium cacodylate buffer. Postfixation was stopped with three buffer rinses (15, 30, 30 minutes) followed by two rinses with ddH_2_O (15, 30 minutes). After dehydration through a graded series of ethanol specimens were embedded in Araldite for TEM and light microscopy. For SEM specimens were critical point dried in a CPD 030 (Balzers Union, Liechtenstein). Dried specimens were sputter coated with gold in a SCD 040 (Balzers Union, Liechtenstein) and viewed with a Fei Quantum 200 scanning electron microscope at 15 kV (FEI Co. Netherlands). Complete longitudinal and transverse serial sections in 0.5 μm thickness for light microscopy of nine specimens from five stages (36 h, 56 h, 96 h, 132 h and 432 h post fertilization (pf)) were sectioned on a Leica Ultracut S. The complete longitudinal sections of one specimen per stage were further used to generate surface rendered 3D-reconstructions. Complete transverse and one longitudinal series of sections in thickness of about 60 nm for TEM were cut from eight specimens from five stages (36 h, 56 h, 96 h, 132 h and 432 h pf). An additional single specimen from stage 432 h pf was serially sectioned alternating between semi-thin sections (0.5 μm) and ultrathin sections (60 nm). Semi-thin sections were stained with toluidin blue. Ultrathin sections were stained with 2% uranylacetate and 2.5% lead citrate in an automatic stainer (NanoWlm Technologie GmbH, Göttingen). Light microscopic images were recorded with a digital camera (Olympus BX-UCB) mounted on an OlympusBX51 compound microscope. TEM pictures were documented with a Philips CM120 BioTWIN electron microscope at 60 kV on Ditabis Digital Imaging Plates were read by a Ditabis Micron IP-Scanner. Images were aligned using open source software Imodalign on a Linux computer [91]. Based on the resulting stack of images 3D-models of the anatomy of all organ systems were created in Amira 3.0 software (Mercury Computer Systems, Berlin). For every developing stage (36 h, 56 h, 96 h, 132 h and 432 h post fertilization (pf)), one 3D-reconstruction on the basis of a complete stack of sections from one specimen was created.

## Competing interests

The authors declare that they have no competing interests.

## Authors’ contributions

SK-S designed and conducted the study. TS collected and fixed the animals. Semithin and ultrathin sectioning was carried out by SK-S. SEM study was done by TS. SK-S generated the 3D-reconstructions and interactive PDFs. SK-S wrote the manuscript. TS contributed constructive criticism on the draft and approved the final version of the manuscript. Both authors read and approved the final manuscript.

## Supplementary Material

Additional file 1: Figure S1 Interactive 3D-PDF of Figure 2. Open with Adobe Reader Version 8.0 or higher. Click here for file

Additional file 2: Figure S2 Internal organization of the late gastrula of *Saccoglossus kowalevskii* (~ 36 h pf). **A** Low mag of the enoderm (*ed*) showing the slit-like central lumen. **B** Sagittal section displaying the area shown in A. **C** Low mag of the anterior endodermal region, i.e. the primordal protocoel (*ppc*). **D** Low mag of the posterior endoderm. The columnar cells are attached to the basement membrane (*arrowheads*). In the area of the former blastopore, no basement membrane is present (in the mid at the bottom of the image). **E** High mag of the apical cell surface of the endoderm. **F** The primordal protocoel is beginning to constrict from the endoderm by means of a sheath of ecm. **G** High mag of the blind ending of the ecm that separates the prospective protocoel from the endoderm incompletely. *ar* archenteron, *ci* cilium, *ec* ectoderm, *ecc* ectodermal cell, *edc* endodermal cell, *mi* mitochondrion, *mv* microvilli, *nn* nerve net, *pcc* protocoelic cell, *vs* vesicles, *yo* yolk. Click here for file

Additional file 3: Figure S3 Interactive 3D-PDF of Figure 4. Open with Adobe Reader Version 8.0 or higher. Click here for file

Additional file 4: Figure S4 Electron micrograph of the early kink stage of *Saccoglossus kowalevskii*. **A** Low mag of cross section of the endodermal tissue. The endodermal cells (*edc*) are highly columnar and filled with numerous granules of yolk (*yo*). **B** The protocoelic lining cells (*pcc*) are differentiated into myoepitelial cells. Longitudinal muscle cells span the protocoel (*pc*) and are connected basally to the basement membrane (*arrowheads*). Additional cells contain basal myofilaments (*myo*) which are orientated circularly. **C** Low mag of the endoderm showing the position of the former connection to the mesocoel outpocking (see 5C for high mag). **D** High mag of a cilium (*ci*) present within the two layers of mesocoelic mesoderm indicating the slit-like lumen (*lu*). **E** Longitudinal section, ventral is down, median is up, anterior to the right.The mesodermal evaginations are separated from the neighbouring tissues by besement membranes. *ec* ectoderm, *ed* endoderm, *msc* mesocoelic cell, *mi* mitochondrion, *mv* microvilli, *za* zonula adherens. Click here for file

Additional file 5: Figure S5 Interactive 3D-PDF of Figure 6 Open with Adobe Reader Version 8.0 or higher. Click here for file

Additional file 6: Figure S6 Interactive 3D-PDF of Figure 8. Open with Adobe Reader Version 8.0 or higher. Click here for file

Additional file 7: Figure S7 Internal organization of the 1 gill slit stage of *Saccoglossus kowalevskii* (~ 132 h pf). **A, B, D-H** transmission electron micrographs. **C** Image of histological sagittal section. **A** Cross section of the developing stomochord (*st*). The single layer of stomochordal cells (*stc*) surround a central lumen (*lu*), and rest basally on a sheath of ecm (*arrowheads*). **B** The stomochordal cells are monociliated and interconnected by zonulae adherentes (*za*). **C** The stomochord protrudes into the protocoel anteriorly (*pc*). The lumen of the stomochord is continuous with the buccal cavity. **D** Sagittal section illustrating the position of the duct of the gill pore (*gp*). **E** Multiciliated cells interspersed between the otherwise monociliated cells lining the buccal cavity. **F** The pericardium (*pd*) is surrounded by a sheath of ecm and contains a central cavity. **G** The anlage of the duct of the proboscis pore (*ppA*) is composed of rather undifferentiated cells at this stage. **H** Podocytes (*po*) are lining the pericardium from the protocoelic side and furthermore rest on a prominent blood sinus (*bs*). *bb* basal body, *bm* basement membrane, *ci* cilium, *ep* epidermis, *mi* mitochondrion, *fe* fenestrations between pedicels, *ms* mesocoel, *mv* microvilli, *myo* myofilaments, *nn* nerve net, *pdc* pericardial cell, *ph* pharynx. Click here for file

Additional file 8: Figure S8 Interactive 3D-PDF of Figure 10. Open with Adobe Reader Version 8.0 or higher. Click here for file
