# Supplementary figures and images for: A detailed description of the development of the hemichordate Saccoglossus kowalevskii using SEM, TEM, Histology and 3D-reconstructions
Source: Front Zool. 2013 Sep 6;10:53. doi: 10.1186/1742-9994-10-53 (PMC4081662; doi:10.1186/1742-9994-10-53)

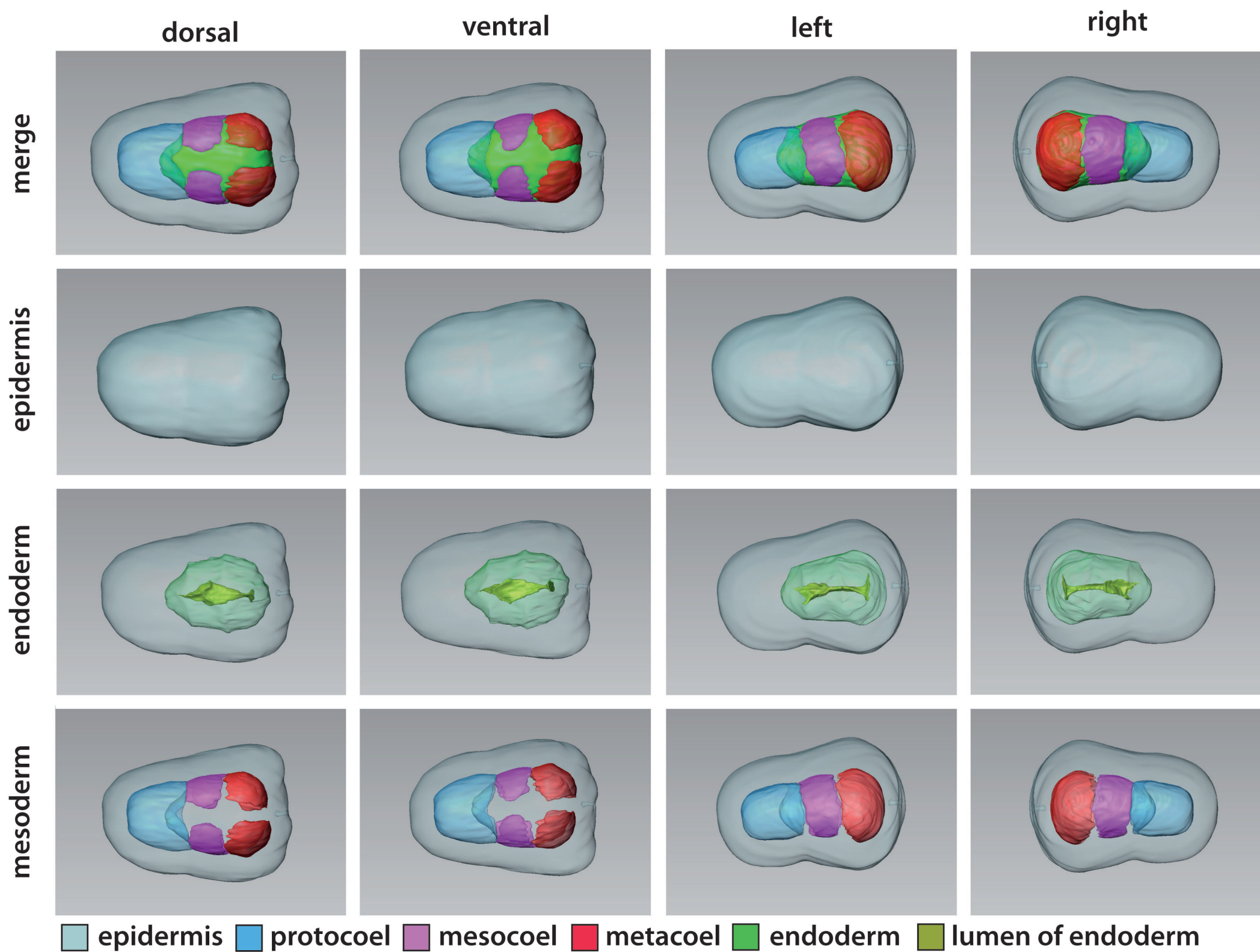

Supplement: Additional file 1: Figure S1 — Interactive 3D-PDF of Figure 2. Open with Adobe Reader Version 8.0 or higher. [file 1742-9994-10-53-S1.pdf]

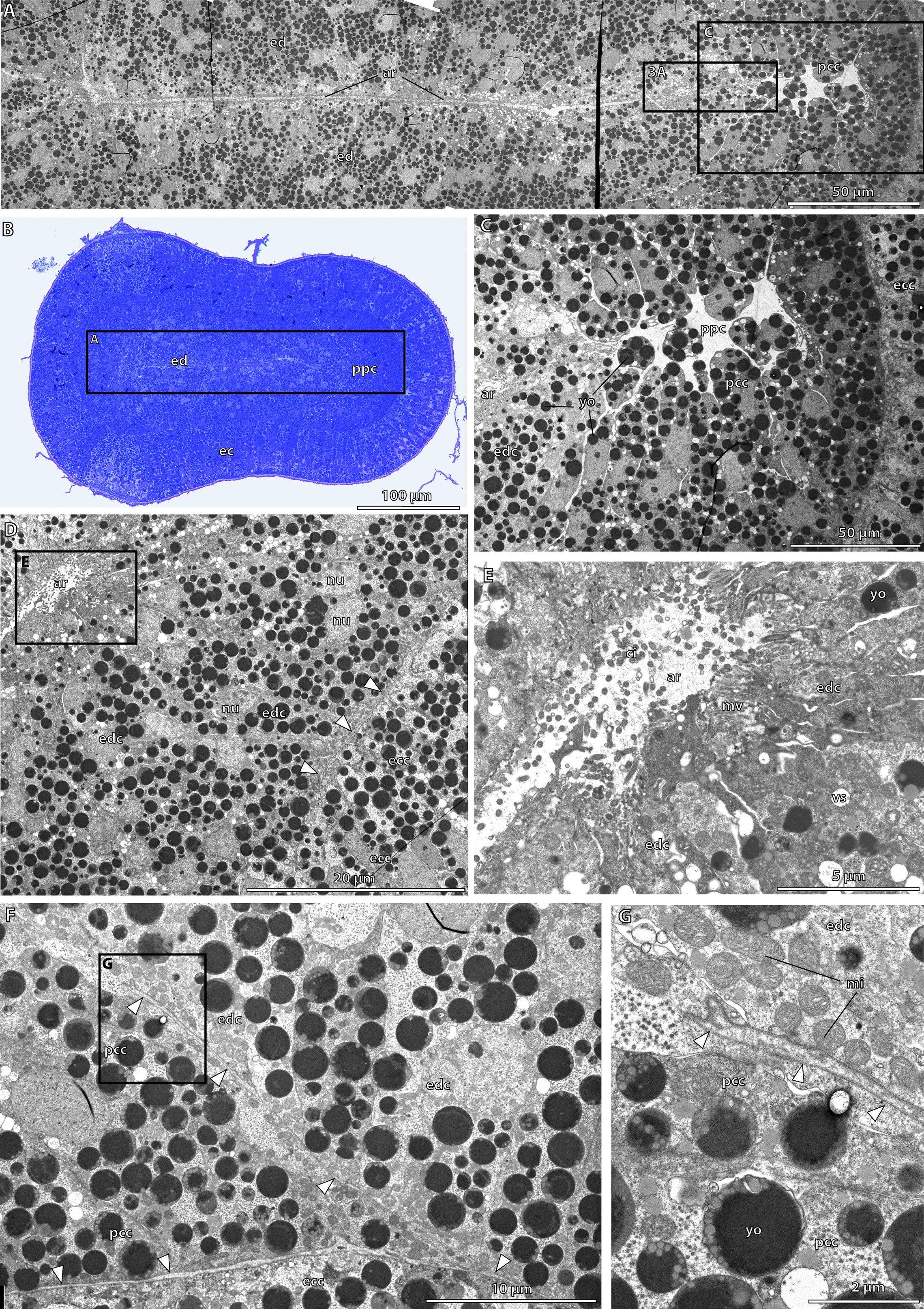

Supplement: Additional file 2: Figure S2 — Internal organization of the late gastrula of Saccoglossus kowalevskii (~ 36 h pf). A Low mag of the enoderm (ed) showing the slit-like central lumen. B Sagittal section displaying the area shown in A. C Low mag of the anterior endodermal region, i.e. the primordal protocoel (ppc). D Low mag of the posterior endoderm. The columnar cells are attached to the basement membrane (arrowheads). In the area of the former blastopore, no basement membrane is present (in the mid at the bottom of the image). E High mag of the apical cell surface of the endoderm. F The primordal protocoel is beginning to constrict from the endoderm by means of a sheath of ecm. G High mag of the blind ending of the ecm that separates the prospective protocoel from the endoderm incompletely. ar archenteron, ci cilium, ec ectoderm, ecc ectodermal cell, edc endodermal cell, mi mitochondrion, mv microvilli, nn nerve net, pcc protocoelic cell, vs vesicles, yo yolk. [file 1742-9994-10-53-S2.tiff]

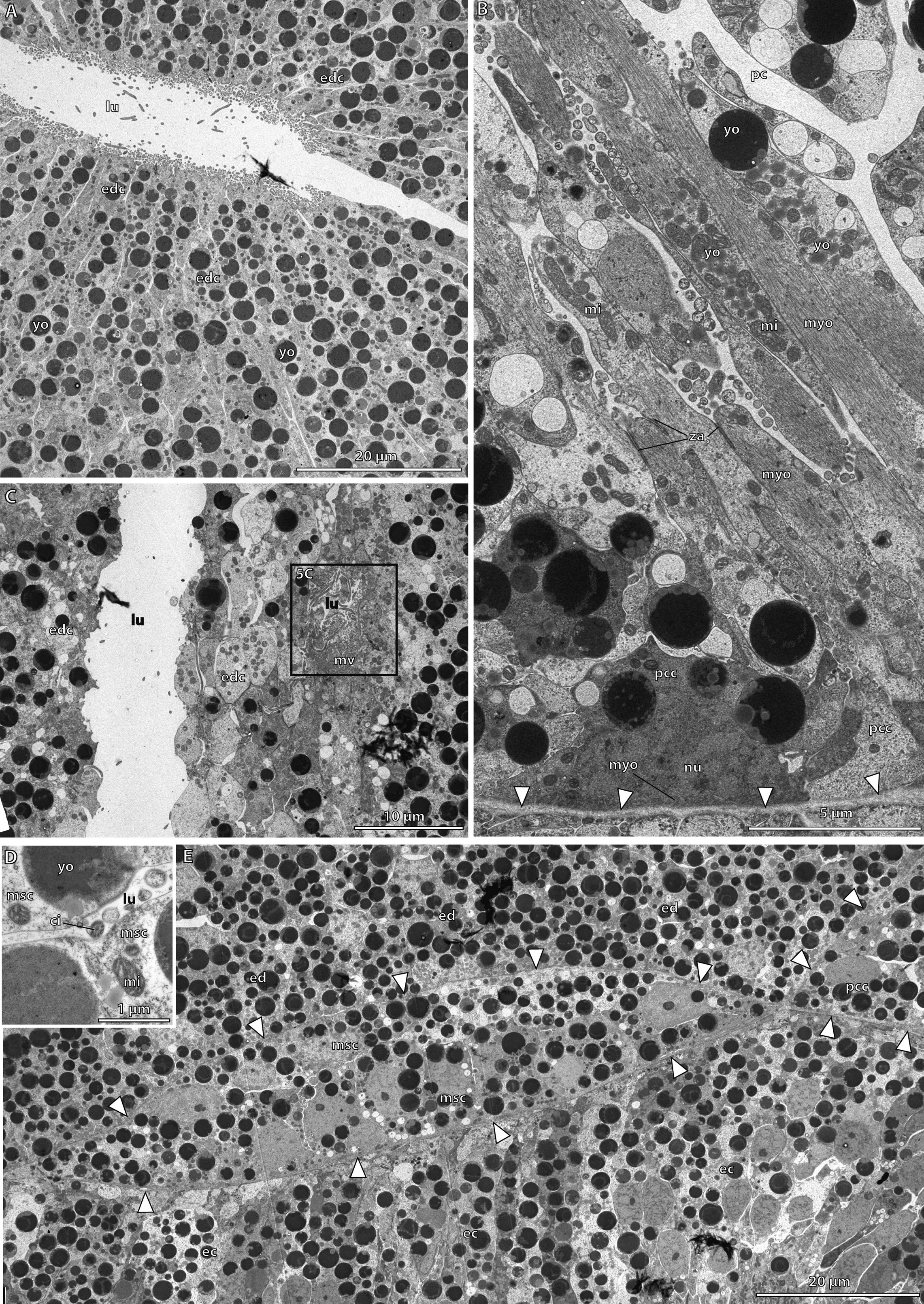

Supplement: Additional file 4: Figure S4 — Electron micrograph of the early kink stage of Saccoglossus kowalevskii. A Low mag of cross section of the endodermal tissue. The endodermal cells (edc) are highly columnar and filled with numerous granules of yolk (yo). B The protocoelic lining cells (pcc) are differentiated into myoepitelial cells. Longitudinal muscle cells span the protocoel (pc) and are connected basally to the basement membrane (arrowheads). Additional cells contain basal myofilaments (myo) which are orientated circularly. C Low mag of the endoderm showing the position of the former connection to the mesocoel outpocking (see 5C for high mag). D High mag of a cilium (ci) present within the two layers of mesocoelic mesoderm indicating the slit-like lumen (lu). E Longitudinal section, ventral is down, median is up, anterior to the right.The mesodermal evaginations are separated from the neighbouring tissues by besement membranes. ec ectoderm, ed endoderm, msc mesocoelic cell, mi mitochondrion, mv microvilli, za zonula adherens. [file 1742-9994-10-53-S4.tiff]

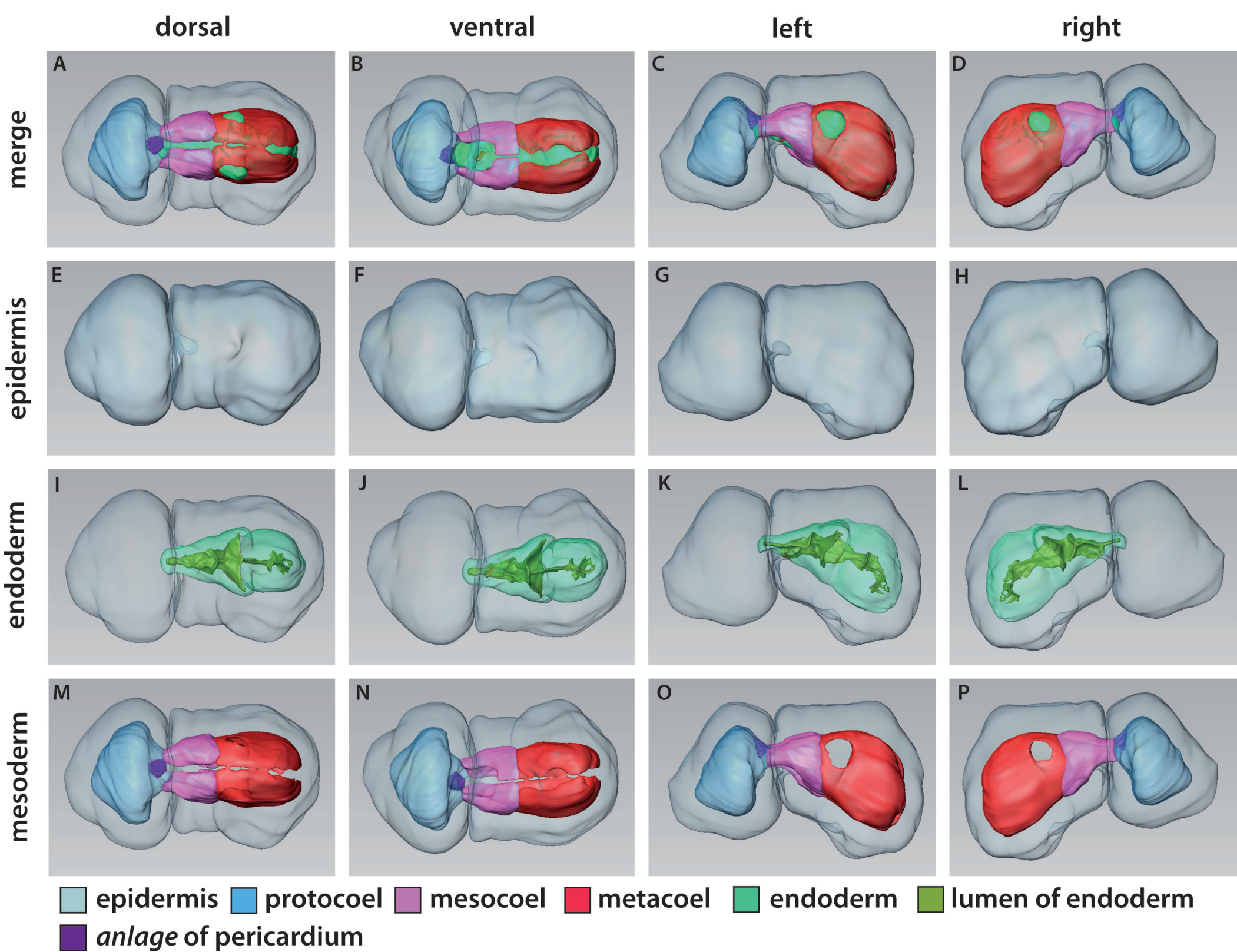

Supplement: Additional file 5: Figure S5 — Interactive 3D-PDF of Figure 6 Open with Adobe Reader Version 8.0 or higher. [file 1742-9994-10-53-S5.pdf]

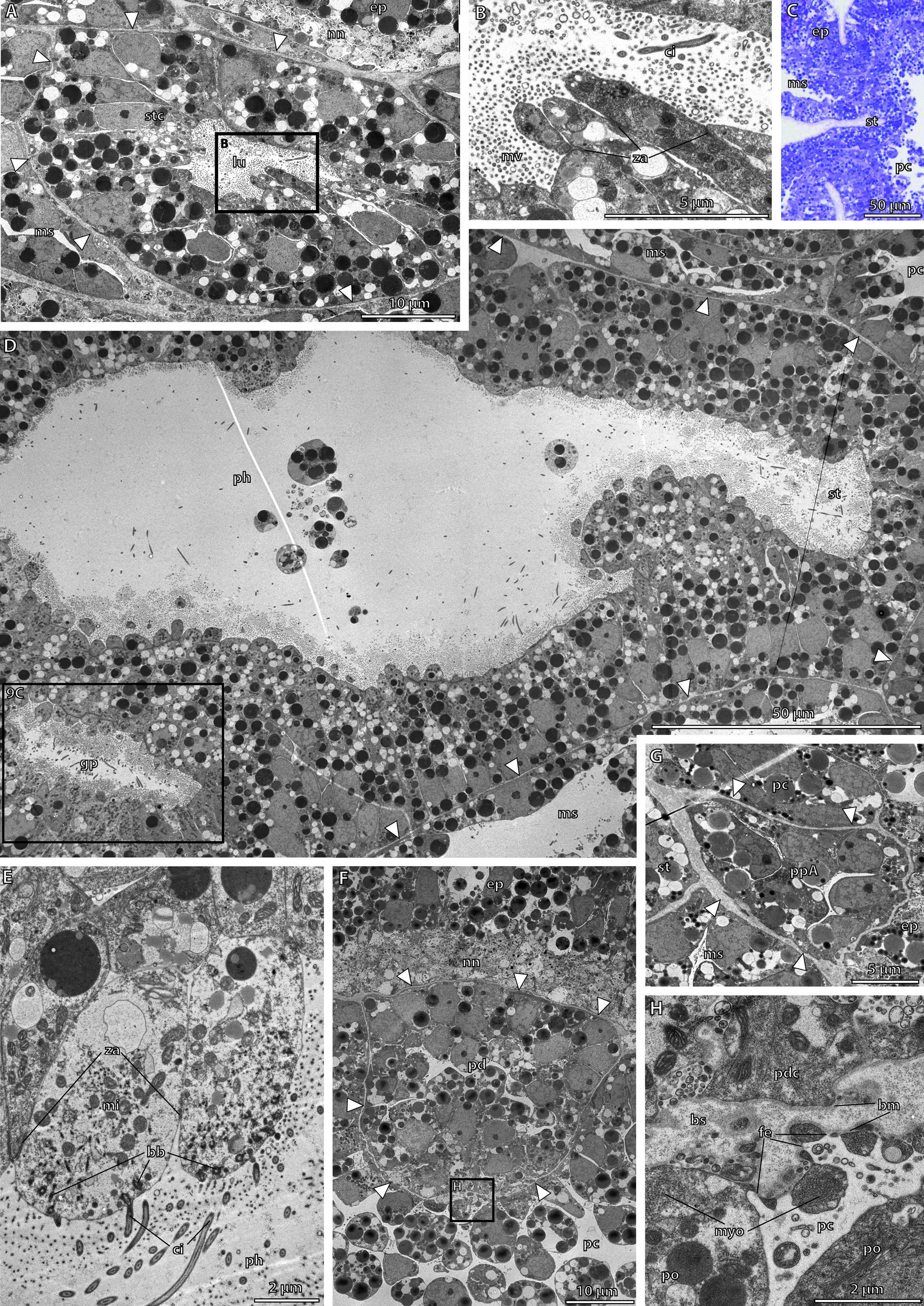

Supplement: Additional file 7: Figure S7 — Internal organization of the 1 gill slit stage of Saccoglossus kowalevskii (~ 132 h pf). A, B, D-H transmission electron micrographs. C Image of histological sagittal section. A Cross section of the developing stomochord (st). The single layer of stomochordal cells (stc) surround a central lumen (lu), and rest basally on a sheath of ecm (arrowheads). B The stomochordal cells are monociliated and interconnected by zonulae adherentes (za). C The stomochord protrudes into the protocoel anteriorly (pc). The lumen of the stomochord is continuous with the buccal cavity. D Sagittal section illustrating the position of the duct of the gill pore (gp). E Multiciliated cells interspersed between the otherwise monociliated cells lining the buccal cavity. F The pericardium (pd) is surrounded by a sheath of ecm and contains a central cavity. G The anlage of the duct of the proboscis pore (ppA) is composed of rather undifferentiated cells at this stage. H Podocytes (po) are lining the pericardium from the protocoelic side and furthermore rest on a prominent blood sinus (bs). bb basal body, bm basement membrane, ci cilium, ep epidermis, mi mitochondrion, fe fenestrations between pedicels, ms mesocoel, mv microvilli, myo myofilaments, nn nerve net, pdc pericardial cell, ph pharynx. [file 1742-9994-10-53-S7.tiff]

dorsal

ventral

left

right

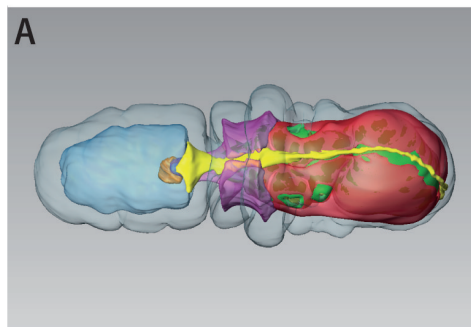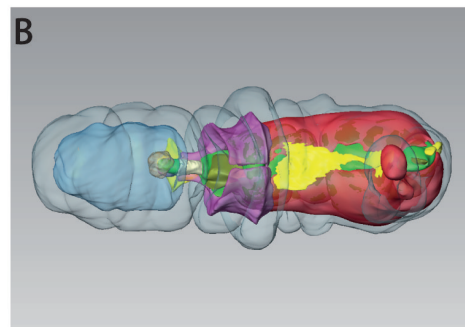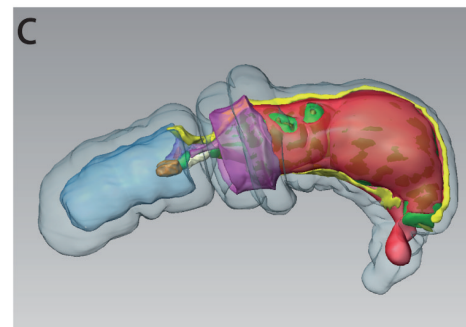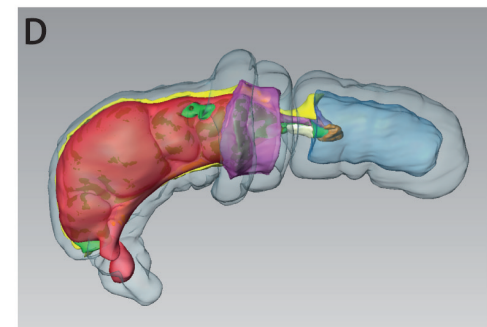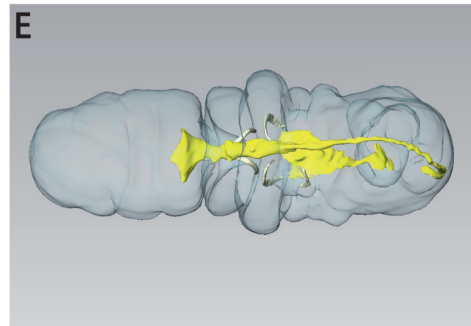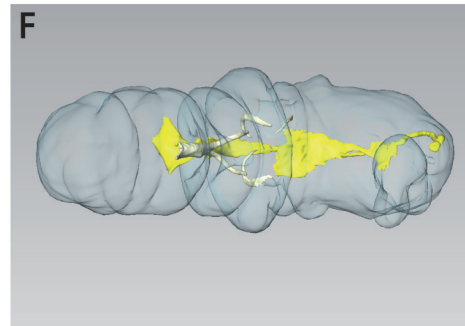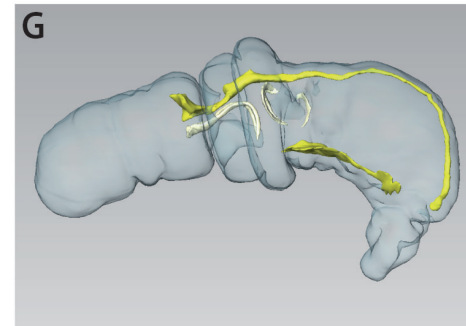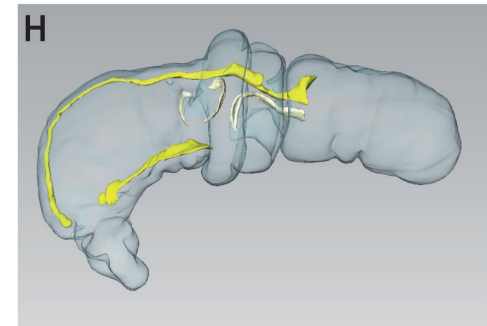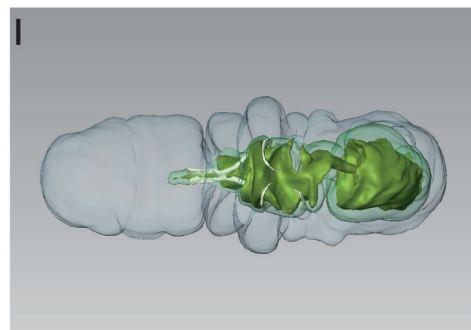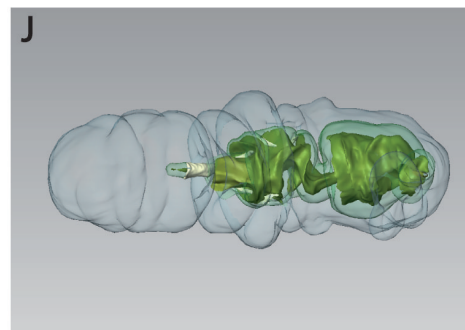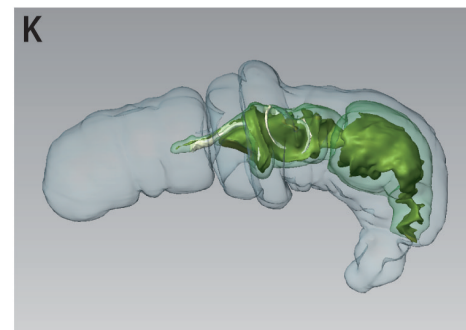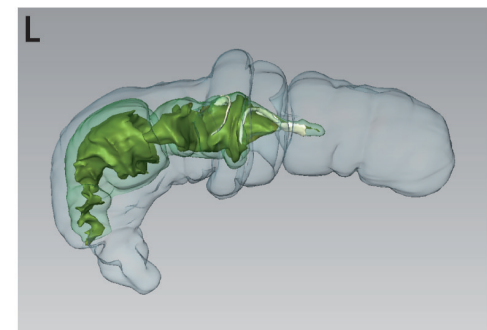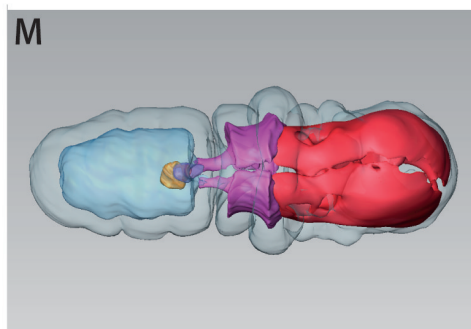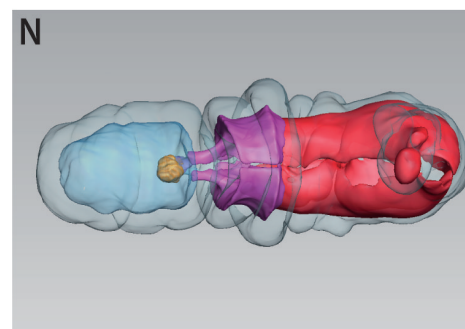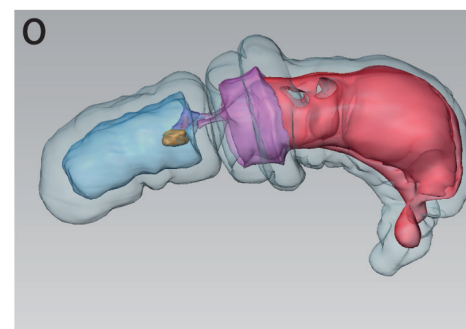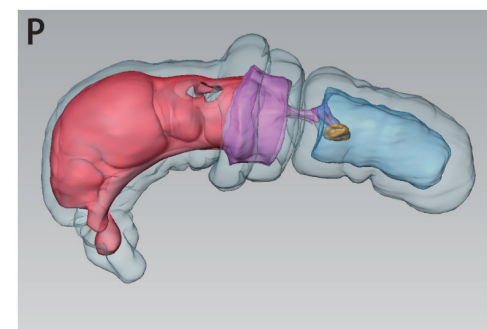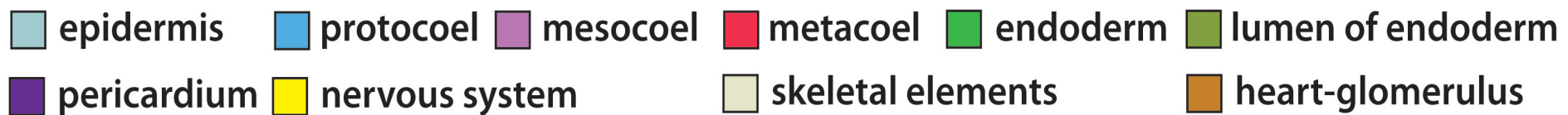

merge

epidermis

endoderm

mesoderm

Supplement: Additional file 8: Figure S8 — Interactive 3D-PDF of Figure 10. Open with Adobe Reader Version 8.0 or higher. [file 1742-9994-10-53-S8.pdf]
