# Supplementary material for: A detailed description of the development of the hemichordate Saccoglossus kowalevskii using SEM, TEM, Histology and 3D-reconstructions
Source: Front Zool. 2013 Sep 6;10:53. doi: 10.1186/1742-9994-10-53 (PMC4081662; doi:10.1186/1742-9994-10-53)
Supplement: Additional file 3: Figure S3 — Interactive 3D-PDF of Figure 4. Open with Adobe Reader Version 8.0 or higher. [file 1742-9994-10-53-S3.pdf]

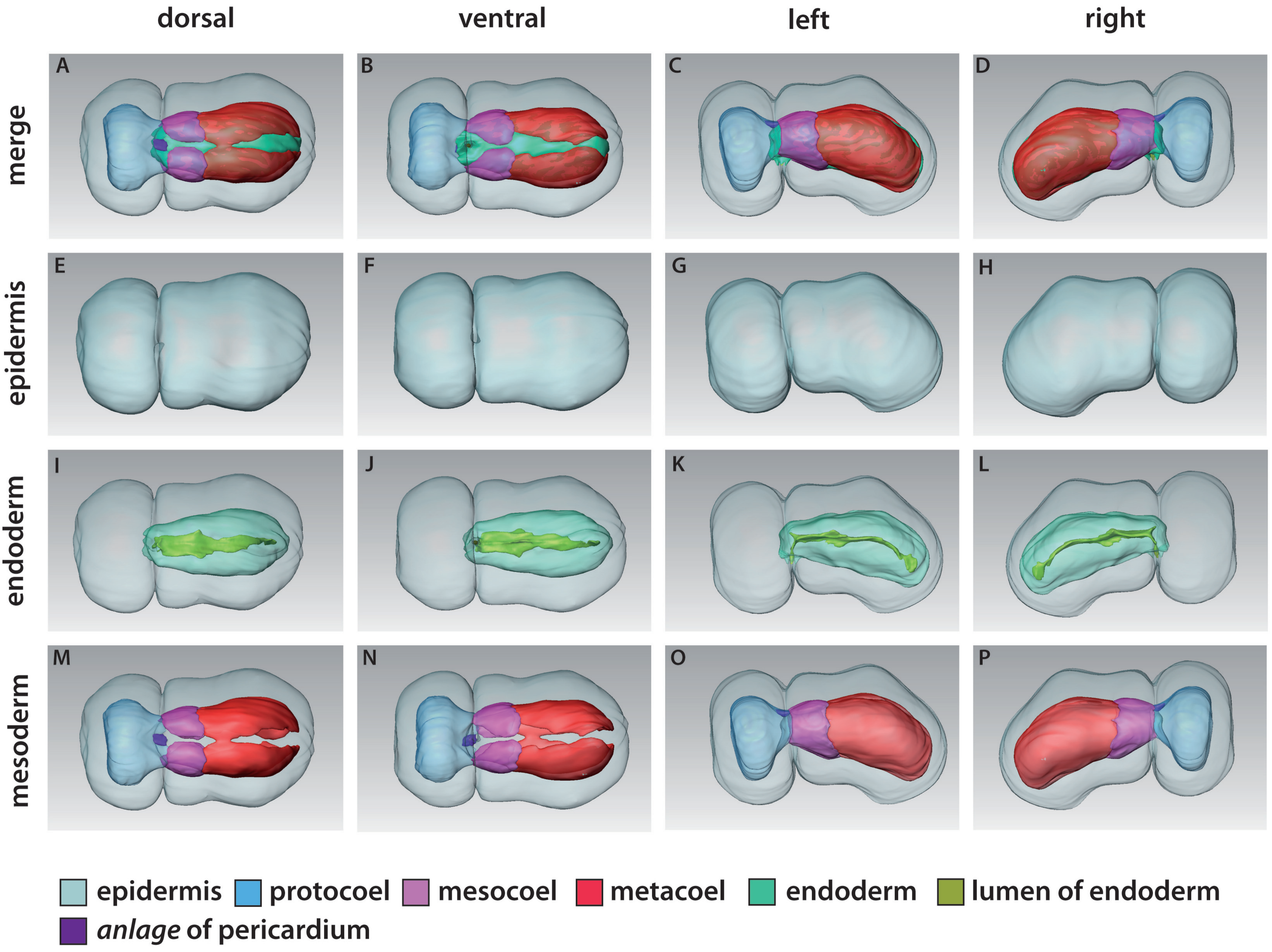
dorsal
ventral
left
right
merge
epidermis
endoderm
mesoderm
A
B
C
D
E
F
G
H
I
J
K
L
M
N
O
P
epidermis
protocoel
mesocoel
metacoel
endoderm
lumen of endoderm
anlage of pericardium
